# Prediction of aqueous intrinsic solubility of druglike molecules using Random Forest regression trained with Wiki-pS0 database

**DOI:** 10.5599/admet.766

**Published:** 2020-03-04

**Authors:** Alex Avdeef

**Affiliations:** in-ADME Research, 1732 First Avenue #102, New York, NY 10128 USA

**Keywords:** aqueous intrinsic solubility, druglike, interlaboratory experimental error, *p*DISOL-X, General Solubility Equation (GSE), Abraham Solvation Equation (ABSOLV), multiple linear regression (MLR), Random Forest regression (RFR), quantitative structure-property relationships (QSPR)

## Abstract

The accurate prediction of solubility of drugs is still problematic. It was thought for a long time that shortfalls had been due the lack of high-quality solubility data from the chemical space of drugs. This study considers the quality of solubility data, particularly of ionizable drugs. A database is described, comprising 6355 entries of intrinsic solubility for 3014 different molecules, drawing on 1325 citations. In an earlier publication, many factors affecting the quality of the measurement had been discussed, and suggestions were offered to improve ways of extracting more reliable information from legacy data. Many of the suggestions have been implemented in this study. By correcting solubility for ionization (i.e., deriving intrinsic solubility, S_0_) and by normalizing temperature (by transforming measurements performed in the range 10-50 °C to 25 °C), it can now be estimated that the average interlaboratory reproducibility is 0.17 log unit. Empirical methods to predict solubility at best have hovered around the root mean square error (RMSE) of 0.6 log unit. Three prediction methods are compared here: (a) Yalkowsky’s general solubility equation (GSE), (b) Abraham solvation equation (ABSOLV), and (c) Random Forest regression (RFR) statistical machine learning. The latter two methods were trained using the new database. The RFR method outperforms the other two models, as anticipated. However, the ability to predict the solubility of drugs to the level of the quality of data is still out of reach. The data quality is not the limiting factor in prediction. The statistical machine learning methodologies are probably up to the task. Possibly what’s missing are solubility data from a few sparsely-covered chemical space of drugs (particularly of research compounds). Also, new descriptors which can better differentiate the factors affecting solubility between molecules could be critical for narrowing the gap between the accuracy of the prediction models and that of the experimental data.

## Introduction

In pharmaceutical research, the aqueous solubility of exploratory compounds is a very important physical property to assess [1, 2]. Peroral drugs with very low solubility may not release sufficient compound from the solid form during the intestinal transit to generate therapeutic benefit. Conversely, highly water-soluble drugs may not be able to permeate lipoidal barriers in the intestinal wall and in the barriers beyond, to reach the therapeutic site of action in sufficient concentration. Thus, not too little and not too much solubility is an important balancing act in compound advancement during drug development.

Given the large number of compounds tested in drug discovery, measurement of solubility is done by high-throughput methods, which generate “kinetic” values in buffers containing 0.5-5%v/v DMSO [2, 3]. Usually, amorphous solids precipitate from supersaturated solutions in the microtitre wells. Although kinetic solubility can be 10-100 times higher than equilibrium solubility, it is nevertheless suitable for anticipating whether a particular test compound will precipitate in an in-vitro bioassay, triggering a false positive test [3-6]. Compounds advanced into later stages of research are fewer in number. Justifiably, more rigorous methods are used to measure their equilibrium solubility, often in media more reflective of the biological fluids to which drugs are exposed [7].

It is beneficial to predict equilibrium solubility of research compounds at the start of discovery projects, as part of virtual screening of compound libraries, before any actual measurements are done, for assisting in the prioritizing molecules for the project. Numerous methods for predicting solubility of organic molecules have been described in the literature, based on quantitative structure-property relationships (QSPR), where the molecular structure is used to predict physicochemical properties [8].

This study concerns prediction of the equilibrium solubility of drugs. Perhaps, more importantly, the focus is on the impact of molecules selected to train the prediction method. The details of the evolving Wiki pS0 database (in ADME Research) [9] of druglike molecules will be described. Since 2011, the focused searching of the primary literature for equilibrium measurements of aqueous solubility (especially as a function of pH) of druglike molecules has contributed to 6355 intrinsic solubility, log *S*_0_, entries. The pre-processing of the available solubility data to extract the underlying *S*_0_ values (normalized to 25 °C [10]) utilized the purpose-designed computer program, *p*DISOL-X (in ADME Research) [11] (whose prototype FORTRAN version, STBLTY, was first coded in the late 1970s [12]). As part of the curation, data quality was assessed by interlaboratory comparisons of those molecules which were studied multiple times by different researchers. The log *S*_0_ values, along with their estimated standard deviations (SD), were then used to train two solubility prediction models: (i) weighted multiple linear regression (MLR) using Abraham solvation descriptors [13], and (ii) Random Forest regression (RFR) [14] using the diverse descriptor collection from the RDKit open-source chemoinformatics and machine-learning library [15]. The results were compared to those calculated by the general solubility equation (GSE), which requires no training [16, 17]. Four external test sets [18-20] were employed in the validation of the models, taking care to remove any of the test set molecules from the large training set. Three of the test sets (containing only druglike molecules) have appeared in landmark ‘Solubility challenges’ [19, 20].

## Methods

### Quantitative structure-property relationships (QSPR) models

#### General solubility equation (GSE)

In 1965 Irmann [21] described solubility prediction based on a group contribution approach. For solids, he included a term related to the entropy of fusion, coupled with the melting point (*T*_m_). In 1968, Hansch *et al*. [22] recognized that the octanol-water partition coefficients, log *P*, are strongly correlated linearly with aqueous solubility values, log *S_w_*, for nonionizable liquid samples. Expanding on the work of Irmann and Hansch, Yalkowsky and coworkers developed and popularized the general solubility equation (GSE), to enable the prediction of solubility of liquids and solids in water [16-18, 23-27]. Just two variables, *T*_m_ (°C) and log *P*, both experimental determined, are used in the equation to predict solubility of organic compounds in water (in log molar units):


(1)

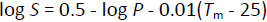



The equation requires no “training.” Although the GSE is rooted in sound thermodynamic principles, some assumptions had to be made in developing the equation: test compounds are taken to be nonionized and fully-miscible in octanol (leading to the 0.5 intercept term), and that the water and octanol phases are assumed not appreciably mutually soluble (but, according to [28]: water-saturated octanol contains ~25 mol% water; solubility of octanol in water is ~2 mM). The implicit assumption behind the 0.01 factor arises from the near constancy of the entropy of fusion. This is in reasonable agreement with the relatively nonflexible aromatic solutes initially considered. A semi-empirical version of the GSE was proposed: the *calculated* log *P* could be used in place of the experimental value. More recently, a version was proposed entirely based on calculated descriptors [27]. Empirically-adjusted coefficients in Eq. (1), based on various training sets [16, 24, 29], did not result in substantially improved predictions of the solubility of druglike substances. The GSE is popular for its ease of use [17].

Yalkowsky and Banerjee [18] proposed an external test set of 21 molecules: 6 solid and 3 liquid poorly-soluble pesticides (log *S*_w_ -3.4 to -7.9), 11 simple drugs (log *S*_w_ 0.5 to -4.1), and a laxative/dye molecule (with somewhat uncertain solubility). As will be shown below (cf., Fig. 11a), the solubility of the above test set molecules is well predicted by Eq. (1). This test set has been widely used by other investigators.

#### Empirical prediction models

Dearden [30], Taskinen and Norinder [31] thoroughly reviewed solubility prediction studies reported from 1992 to 2005 [25, 29, 32-47] which used the popular Yalkowsky-Banerjee external test set to assess the efficacy of the empirical methods. The average of the reported prediction root-mean square errors (RMSE) is about 0.9 log unit, with individual values found to range from 0.6 to 1.4. The predictions of Raevsky *et al*. [29] (nearest-neighbor method, using HYBOT hydrogen bond descriptors) and Tetko *et al.* [40] (artificial neural network method, with electrotopological E-state indices) fared slightly better than those of others. Many of the training sets used in the prediction studies consisted of several hundred simple organic molecules, including aromatic hydrocarbons, polyhalogenated organic compounds, practically-insoluble agrochemicals and environmental pollutants, many in liquid form at room temperature, but only relatively few druglike molecules (resulting in spotty coverage of the chemical space resembling today’s pharmaceutical discovery compounds). As summarized in the reviews [30, 31], prediction methods included multiple-linear regression (MLR), principal components regression (PCR), partial least-squares (PLS), k-nearest neighbors (kNN), artificial neural networks (ANN), support vector regression (SVR), and Random Forest regression (RFR). Some of the QSPR methods were based on hundreds of calculated atomic and molecular 2D and 3D descriptors. In many of the studies, the most influential descriptors are two calculated physical properties: log *P* and molar refractivity, *MR*, (which accounts for molecular size and polarizability). Other calculated 2D descriptors included partial-charge surface properties, atom and functional group counts, connectivity and topological and electrotopological indices, H-bond donor and acceptor counts; 3D descriptors included energy terms (total potential energy, electrostatic, molecular mechanics force-field energy), molecular shape, volumes, and water-accessible surface areas [48-55].

Wang and Hou [56] summarized solubility prediction efforts up to 2010, comparing the results of 16 studies. They discussed the improvements resulting from consensus modeling. Also, there was a discussion of using “local data” models to improve predictability, with the domain of applicability (DOA) identified by molecular descriptor similarity, rather than structural (*e.g.*, Tanimoto indices) similarity.

#### Abraham solvation equation (ABSOLV)

Abraham and Le [13] amended the Abraham solvation equation [57] to predict solubility:


(2)

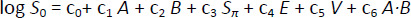



In the MLR equation, the log *S*_0_ is the dependent variable (measured log intrinsic molar solubility) and the independent variables are the five solute descriptors accounting for the transfer of solute from one phase to another: *A* is the sum of H-bond acidity, *B* is the sum of H-bond basicity, *S*_π_ is the dipolarity/polarizability (subscripted here, so as not to be confused with solubility), *E* is an excess molar refraction in units of (cm^3^∙mol^-1^)/10, and *V* is the McGowan characteristic volume in units of (cm^3^∙mol^-1^)/100. The c_0_-c_6_ coefficients in Eq. (2) are determined by MLR, trained on a set of intrinsic solubility values of a diverse collection of molecules. The five Abraham solvation descriptors may be calculated from 2D structure (introduced as a SMILES text or as coordinates in a ‘mol’ or ’sdf’ type file) using the program ABSOLV [58] (*cf*., www.acdlabs.com). The *A∙B* cross-term in Eq. (2) is intended to deal with intermolecular H-bond interactions between acid and base functional groups in the solid or liquid environment. Its inclusion, as an alternative to using the *T*_m_ term in Eq. (1), was intended to improve the prediction accuracy. Eq. (2) applied to the Yalkowsky-Banerjee external test set, using the MLR coefficients reported by Abraham and Le (their Eq. 11), with ABSOLV-calculated descriptors, resulted in RMSE = 1.71 log unit (prostaglandin-E2 was an extreme outlier; data not shown). In the present study, we re-determined the seven MLR coefficients using our own training data, with the data weighted according to estimated measurement errors, to find a much better fit, as will be shown below (cf., Fig. 12a).

#### Random Forest regression

Of the new machine-learning statistical approaches, the Random Forest regression (RFR) method is thought to be among the top performers, in terms of prediction accuracy. The method was introduced in 2001 by Brieman [14], and is implemented in the open-source “randomForest” library for the R statistical software [59-61]. RFR may be appealing to new users because it can be employed “off the shelf,” requiring only minimal learning. In many applications, the default “tuning” parameters are nearly optimal. RFR works by constructing an ensemble of hundreds of decision trees [62].

**Figure 1.**
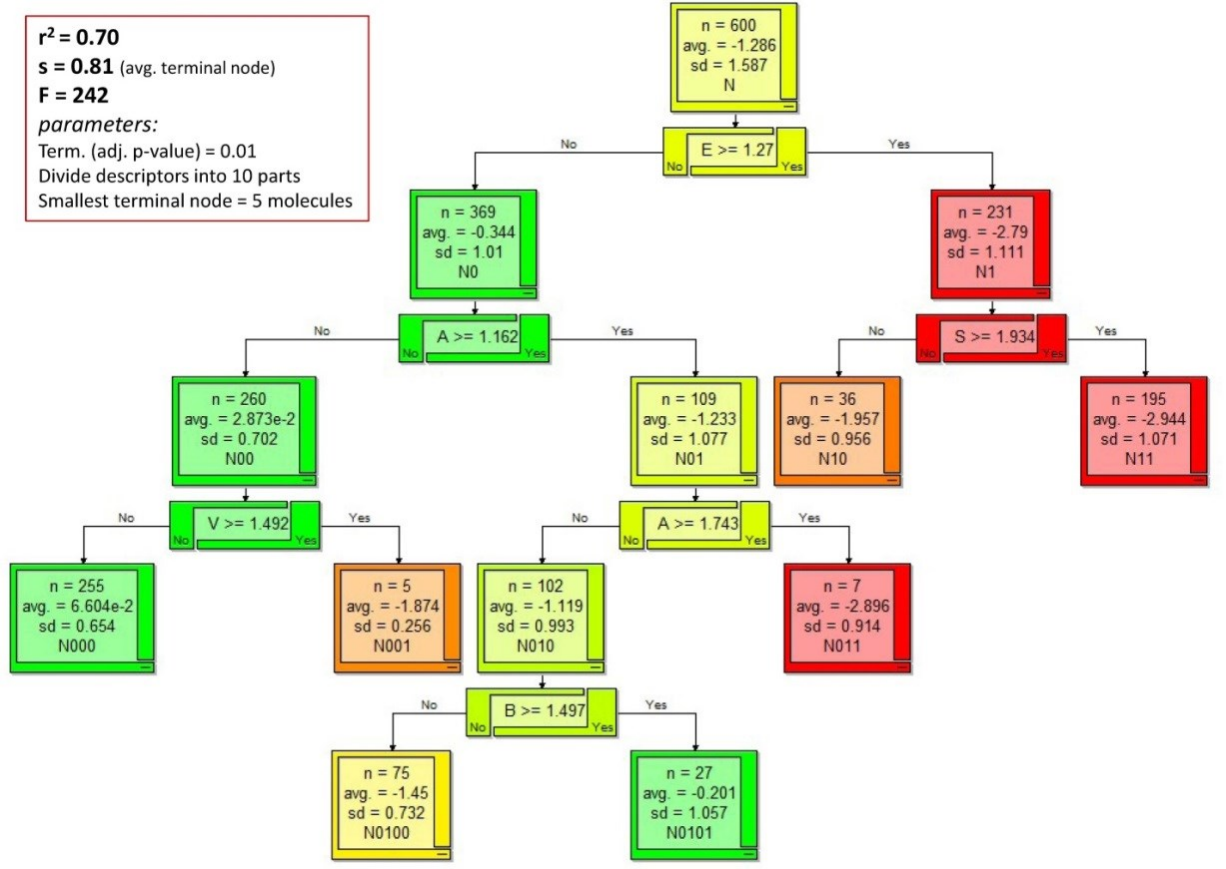
Example of a calculated recursive partition decision tree (Algorithm Builder v.1.8), based on 600 zwitterionic molecules (*Wiki-pS_0_* database), using Abraham descriptors. At each node, all five descriptors are queried to select the one best suited for further splitting of the data. In part, node splitting stops at 5 molecules. By comparison, the Random Forest method uses hundreds of trees (each containing a different subset of randomly-selected solubility values of molecules) and re-selects a subset of descriptors randomly for each node splitting

To illustrate, in part, how RFR works, Figure 1 shows an example of a *single* recursive partition decision tree constructed (Algorithm Builder v.1.8, ACD/Labs, Toronto, Canada; www.acdlabs.com), using the 600 zwitterionic molecules in the *Wiki-pS_0_* database, drawing on the five Abraham descriptors [57]. The process begins with the unsupervised selection of one of the descriptors (*E* in the example) and finding the optimal ‘splitting’ value (1.27 in the example) which divides the solubility data into two branches: the left branch grouping 369 molecules which have descriptors less than the splitting value and the right branch grouping 231 molecules with descriptors equal to or exceeding the splitting value. A criterion for the splitting can be based on minimizing the residual sum of squares at each node,


(3)

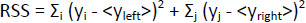



where i indexes the solubility values in the left branch and j indexes those in the right branch; y represents log *S*_0_ values; <y> is the average value in the left/right branch. Each of the two branches generates a new node. The process then repeats until the “terminal” nodes are reached, associated with a specified minimum of molecules (*e.g.*, 5). In the above decision tree training, r^2^ = 0.70 and RMSE = 0.81 (average of the seven terminal “leafs”). Generally, the node splitting procedure yields ever more homogeneous groupings of molecules, and produces trees which bring together similar solubility values at the same node.

The above example involved just one tree, where at each node, *all* of the descriptors were considered in the selection of the one best suited to split the node. RFR is different in a number of ways. Typically, 500 decision trees – a “forest” – are constructed.

Liaw [61] graphically illustrated the structure of a typical random forest. The entire data matrix comprises n rows of solubility values and p columns of chemical descriptors. Each tree in the forest is allocated a different bootstrap (with replacement) sample of the n rows – *i.e*., it contains a randomly-selected subset (*e.g*., two thirds) of the entire solubility data. For each tree, the “left out” molecules (*e.g.,* one third) are called the ‘out-of-bag’ (OOB) sample. Each tree is grown to its maximum size by node splitting, as partly illustrated in Figure 1. In RFR, only a randomly-selected subset of the available descriptors (typically, p/3) is used at each node in each tree. Each tree is grown until the terminal nodes are reached, with each final “leaf” containing a specified minimum number of solubility values, the average of which being the predicted value for the particular tree. The final prediction for the regression model is made by averaging predictions from all trees. All the compounds that did not take part of the tree growing process (OOB compounds) can be used as an internal validation set to estimate the error of the model.

To assess the predictability of the models in the current study, we randomly split the solubility data into a training set (70%) and an *internal* test set (30%), as described by Walters [63]. Also, *external* test sets proposed by others were predicted based on the RFR model trained with all of the molecules (excluding any from the external test sets).

RFR is not sensitive to the presence of irrelevant descriptors, even those which are highly correlated. Hence, “over-fitting” the data is not expected. (However, it is noteworthy that if test set molecules are also included in the training set, then their RFR “prediction” will be very close to the user-provided *measured* values.) RFR includes built-in estimation of (i) prediction accuracy (as standard deviation of the predicted mean), (ii) descriptor importance (as a result of sensitivity testing of each descriptor), and (iii) similarity between molecules (as a result of the node filtering process). The application of the method to QSAR predictions has been described in detail by Svetnik *et al.* [64]. An inconvenience of the currently-developed RFR method is that it cannot *extrapolate* (in the sense that MLR methods can): it cannot predict any solubility value *outside* of the range encompassed by the training set. For example, the extremely-low (log *S*_0_ < -8) solubility of drugs like *amiodarone*, *clofazimine*, *itraconazole*, *halofantrine*, and *probucol* is not expected to be well estimated by RFR. The latter molecules are near the edge of the chemical space (defined by the descriptors used) that’s sparsely populated by molecules with similar solubility. The closest molecules are likely to be more soluble than the above test compounds.

The first applications of RFR to predict solubility appeared in 2007 [65, 66]. Schroeter *et al.* [65] used *S*_w_ and *S*_pH_ data (mixed values not corrected for ionization) to train a RFR method, using ~4000 measurements mostly taken from secondary sources [35, 67, 68] and some from in-house (Bayer Schering Pharma) sources. For the Huuskonen data [35] as test set, RMSE = 0.66 (n=1290) was reported. For the solubility data in the domain of applicability (DOA) matching that of research compounds (10^-3^ to 10^-7^ M solubility), the RFR method indicated RMSE ~ 0.85 log unit. In the Palmer *et al.* [66] RFR analysis, aqueous solubility values of 998 structurally diverse druglike solid organic compounds were gathered from similar secondary sources: *Handbook of Aqueous Solubility* [69], Huuskonen [35], and Delaney [47]. (It was not reported how molecules were corrected for ionization.) The authors used the molecular operating rnvironment (MOE) [70] to generate 126 two-dimensional (log *P*, *MR*, charged-surface properties, atom, group, and H-bond counts, connectivity and topological indices) and 36 three-dimensional (total potential energy, electrostatic contributions, molecular shape, and solvent-accessible surface area) descriptors. Various values of the RFR tuning parameters, *ntree*, *mtry*, and *nodesize,* were explored in the model trained with all of the 2D descriptors, with the best parameter values found to be *ntree* = 500, *mtry* = 42, and *nodesize* = 5, which are the usual default values. The training set of compounds produced the statistics: r^2^ = 0.98, RMSE = 0.28, n = 988, bias = 0.007. As often pointed out, this is not an accurate measure of the predictability of solubility of molecules not used in the training process. Randomly splitting the entire data into a training set (70%) and an internal test set (30%) produces a good measure of the ability of the model to predict solubility of compounds not included in the training set, indicated r^2^ = 0.89, RMSE = 0.69, n = 330, bias = 0.017. An external test set produced similar statistics. Including the 3D descriptors did not make substantial improvements to the model.

The most influential descriptors in the Palmer *et al.* study were calculated to be those related to the fractional van der Waals surface area, *VSA*. The ten most important descriptors ranked by RFR were log *P* > negative VSA (*PEOE_VSA_FNEG*) > number of hydrophobic atoms (*a_hyd*) > *MR* > hydrophobic atoms VSA (*vsa_hyd*) > *chi1v* (topological) > polar VSA (*PEOE_VSA_FPOL*) > hydrophobic VSA (*PEOE_VSA_FHYD*) > *MW* > negative polar VSA (*PEOE_VSA_FPNEG*).

More recently, Walters [63] thoroughly compared the Huuskonen thermodynamic *S*_w_ values (n = 1274) [34, 35], the Llinas *et al.* thermodynamic *S*_0_ values (n = 94) [19] and PubChem (n=1000) kinetic high-throughput solubility [71] databases using the RFR framework. The publication serves as a very useful tutorial to the machine-learning method, and is highly recommended for those interested to try RFR.

### Gap between prediction and experiment

For 411 compounds characterized by multi-source solubility measurements, Katritzky *et al.* [72] found standard deviation, SD, to be 0.58 log in replicate values. According to Taskinen and Norinder [31], an AstraZeneca in-house database of solubility measurements of different batches of the same compound typically showed reproducibility of 0.49 log. Higher uncertainties had been discussed (Jorgensen and Duffy [73]; Palmer and Mitchell [74]). It has been a widely-shared view that interlaboratory *measurement* reproducibility is typically 0.6 log.

As mentioned previously, the solubility *prediction* errors are often in the 0.6 to 1.3 log unit range [30, 31, 56, 73, 74]. So, one might surmise that prediction methods are approaching measurement error limit. But, this may not be so.

First, many of the early prediction studies considered molecules from a chemical space occupied by relatively simple organic molecules and some complex agrochemicals, which were adequately represented by the then available training set data. In some of these studies, low RMSE were achieved. Earlier training sets were under-represented in practically insoluble and highly lipophilic *druglike* molecules, whose physicochemical properties are not easy to measure accurately. In some cases, important descriptors, such as *calculated* log *P* can be off by 1-2 log units (*e.g.*, amiodarone). Since values of log *P* > 5 or < -2 are difficult to measure accurately by the shake-flask method [28], log *P* prediction methods can be uncertain for out-of-bounds molecules. At such extreme values, experimental log *P* values may not strongly correlate with the experimental log *S* values [75]. Since many of today’s research compounds have very low solubility, the earlier prediction methods that have shown low RMSE are not expected to do as well when subjected to predicting solubility of practically insoluble drug molecules, such as *amiodarone* and *itraconazole*, or novel research compounds synthesized in drug discovery programs, for which there may be a shortage of prediction training set data publically available.

Second, the perceived 0.6 log error in *measured* solubility may be upwardly biased, given how disparate legacy data have been handled in assembling large training sets. The relatively poor reproducibility may be the result of systematic errors arising from mixing different types of solubility values, measured at different temperatures, or simply gathered from poor-quality measurements. A ‘white paper’ drawing on expert consensus thoughts of researchers from six countries addressed the critical needs related to experimental assay design, and *how legacy data can be better processed to reveal improved precision* [76]. A related study [9] discussed at length the correction of data for ionization when solution complexity distorts the expected shape of the log *S-*pH profile predicted by the Henderson-Hasselbalch equation. When solubility values measured in the temperature range 10-50 °C are transformed to values at 25 °C, the estimates of the interlaboratory precision improve [10].

The above two points suggest that the gap between prediction and experimental errors may still be substantial. Similarly, Palmer and Mitchell [74] made the case that it’s not the data that are limiting, but rather it’s the prediction methods (and/or descriptors) that need further improvements. In an earlier review, Faller and Ertl [77] suggested that “no really satisfactory approach to [drug] solubility prediction is available yet,” in spite of the large number of prediction studies.

### Quality and chemical space of experimental data

It has been consistently shown that the best prediction models are devised from training set molecules that occupy very similar chemical space (defined by the descriptors used) as those in the test set [63]. For drug solubility prediction, the ideal training sets would consist of molecules of interest to discovery projects. Only a tiny fraction of such measurements are publically available, and in-house pharma prediction studies are unlikely to be openly publicized.

Measuring equilibrium solubility of ionizable molecules is expensive and analytical-resource consuming. Even given high analytical investment, quality is not assured when results are based on poorly-designed assays.

### Factors affecting reproducibility in published solubility data – ‘white paper’ summary

Many of the factors affecting the quality of equilibrium solubility measurement have been discussed in the consensus report (‘white paper’ [76]) are summarized in the list:

- dissolution of added solid has not reached equilibrium during the selected equilibration time,
- solid state characterization not performed after equilibration - polymorphs, hydrates, solvates, nanoparticles, amorphous forms not identified,
- formations of drug aggregates/oligomers (dimers, trimers, …), micelles, and drug-buffer complexes in solution at equilibrium [78],
- poor wettability,
- adsorption to filter/vial surface,
- inappropriate phase separation methods used, *e.g.,* (i) first centrifuging a saturated solution, then filtering the supernatant (without first saturating the filter); (ii) multiple re-centrifuging a centrifuged solution (without pre-saturating the vial surfaces); (iii) nano-sized particles passing through filter,
- using unnecessarily high buffer concentrations, possibly effecting drug-buffer complexation [78],
- not using buffers with low-soluble ionizable drugs (especially weak bases),
- effect of impurities unaccounted, especially those which are ionizable when unbuffered solutions are used,
- not measuring the final pH of the equilibrated saturated solution of ionizable drugs (buffered pH may be altered by the drug),
- not taking into account the effect of ambient CO_2_ on the water solubility of low-soluble bases in unbuffered solutions,
- inadequate pH electrode calibration at low/high pH (junction/asymmetry effects), and in drug-salt studies (high ionic strength),
- compound instability at the extremes of pH or over long saturation times (*e.g*., indomethacin, acetylsalicylic acid, ascorbic acid),
- stereoisomers (DL-, D-, L-), (R-/S-), or cis-/trans-isomers not stated,
- limit of detection (LOD) - not sufficiently sensitive analytical methods used to determine drug concentration below LOD,
- for ionizable compounds, inaccurate value of p*K*_a_ used to calculate log *S*_0_ from log *S*-pH profile introduces systematic error.

The impact of the above factors can be minimized by employing good experimental practices and appropriate data analysis methods. However, in today’s solubility prediction methods, factors such as the formation of differing polymorphs, hydrates, solvates, amorphous solids, and the impact of stereoisomers, are not adequately addressed.

## Data

### Wiki-pS_0_ database

The intrinsic solubility database, *Wiki-pS_0_* (*in-ADME* Research), contains 6355 log *S*_0_ (log molar) entries, based on measured aqueous solubility values of 3014 different compounds collected from 1325 cited references (as of April 2019). In the majority of the cases, the literature data were further processed, using *p*DISOL-X (*in-ADME* Research), to extract intrinsic solubility (*S*_0_) values from reported aqueous free-acid/base or salt solubilities (*S*_w_), solubilities at specified pH (*S*_pH_), or log *S*-pH profiles [9, 11, 76, 78-81]. All of the molecules are solids at room temperature (except for propofol, whose *T*_m_ is 14 °C). There are 1078 log *S*_0_ entries derived from 9907 individual log *S* measurements at a particular pH (cf., Fig. 1a in [9]). About half of the data sources originate from secondary listings and the rest are from primary sources. In the case of secondary sources, the citations to the original work are generally available, and in many cases were consulted for clarifications. Differently named molecules were identified and reconciled by searching the database for matching Tanimoto structural fingerprint indices [15].

For 3671 entries, comments were added to the database records (based on available information in the original sources), briefly noting experimental method used (mostly saturation shake-flask), temperature (23 °C assumed when ‘room temperature’ was stated or no value was provided), equilibration time, apparent quality of data, standard deviation in measured values (if reported), buffers/pH, polymorphic or hydrate form (if identified), method of solid separation, agitation method, etc.

The most reliable data had been determined by the saturation shake-flask (SSF) method (still the “gold standard” methodology in the minds of most experimentalists), especially when taken *as a function of pH*. Also, two potentiometric instruments have demonstrated their importance: pSOL [82] and CheqSol [83] (both now available from Pion Inc., Billerica, MA, USA). The characterization of solid forms (crystalline, amorphous, nanoparticle, etc.) and their impact on the measured solubility are important considerations (i.e., solvate, polymorph, racemate effects), but these are not always reported/detailed in the solubility studies.

Two websites: ChemSpider (Royal Society of Chemistry, UK) www.chemspider.com, and PubChem (https://pubchem.ncbi.nlm.nih.gov/ were valuable for checking names of molecules, obtaining CAS numbers, getting structure representations (SMILES), melting points (*T*_m_), and the like. ACD/Labs ChemSketch was useful for drawing molecules and constructing SMILES representation for molecules. When measured *T*_m_ were not found (as in 19% of the entries in *Wiki-pS_0_*), then Lang and Bradley [84] *predicted T*_m_ were used: QsarDB open repository of data and prediction tools (http://qsardb.org/repository/predictor/10967/104?model=rf).

#### Data added to *Wiki-pS_0_* from multi-source compilations (‘low hanging fruit’)

- PHYSPROP database [67] (Sept 1999 version: over 6000 measured water solubility, *S*_w_): 1327 values were selected for molecules not appreciably ionized in water. Excluded compounds were: (a) *T*_m_ < 40 °C, (b) log *S*_w_ < -8 or > 0, (c) surfactants/long aliphatic chain molecules, (d) polycyclic aromatic hydrocarbons, (e) peroxides, (f) carboxylic acids, (g) salts/complexes, (h) dyes or names containing color, and (i) herbicides, pesticides, insecticides, rodenticides, and acaricides (as indicated by “tags” at the ChemSpider website). Of the selected 1327 compounds, the *S*_w_ values of 1210 nonionizable/nonionized molecules were taken to be *S*_0_. The other 117 compounds were processed by *p*DISOL-X to calculate *S*_0_ and pH_sat_ (pH of saturated solution) from the given *S*_w_ and p*K*_a_, assuming pure water was the solvent, and the Henderson-Hasselbalch equation was valid [11]. Literature references (many from Merck Index and Beilstein – *cf*., below) were recorded in *Wiki-pS_0_*.
- *Handbook of aqueous solubility data* [85]: 1130 *S*_w_ of druglike molecules were selected, with 776 values subjected to *p*DISOL-X analysis to determine *S*_0_ values. Some values were listed as intrinsic in the handbook, only requiring adjustments when the temperature was not 25 °C. Original references were recorded in *Wiki-pS_0_*. Many references were checked; however, references for 65 compounds could not be accessed online. Occasionally, reported *S*_w_ values for neutral compounds were actually those of drug-salt measurements, as clarified on checking the original literature.
- *Beilstein* [68] (*cf.*, [67]): *S*_w_ values of 474 compounds were used, after conversion to the *S*_0_ scale, where necessary.
- *Benet-Broccatelli-Oprea 927 BDDCS solubility list* [86]. This compilation contains interesting drugs, but no references to original sources were cited and no experimental details were given. Of the drug solubilities listed, 333 were selected. In many cases, the original sources were recognized on cross checking with existing entries in *Wiki-pS_0_*. The *S_w_* values were mostly of free bases/acids, but some were clearly of salts, which required careful effort to discern. All selected values were converted to the *S*_0_ scale using *p*DISOL-X.
- *Analytical profiles of drug substances (APDS)* [87]. The first 39 volumes of the series of monographs were searched for quantitative solubility data. Monographs on 155 molecules were selected for pre-processing. Most of the reported solubility values of ionizable molecules were measured in pure water with unspecified saturation pH. For those ionizable molecules which were not drug salts, the intrinsic values were calculated by *p*DISOL-X. Unfortunately, the solubility reported in APDS is often devoid of experimental detail (*e.g.*, temperature not always reported), some citing ‘personal communication’ as references. Nevertheless, there are several high-quality log *S* - pH original data sets in the monographs.
- *Merck index* [88]. *S*_w_ values of 173 molecules were used, after conversion to the *S*_0_ scale. The Merck Index is often cited in older databases (*e.g*., [67]), but it may not be a sufficiently reliable general source for critical studies (literature references not usually given, details often lacking, etc.).
- *Biowaiver monographs for immediate release solid oral dosage forms* [89]. Dressman and colleagues published a series of papers (2005-2018), from which 14 drug solubility values were added to *Wiki-pS_0_*, some being not previously-published measurements.
- Miscellaneous collections: Freier’s book [90] - 96 values were selected; *Handbook of Biochemistry* [91] - 54 values were used; Kühne *et al.* tabulation [33] - 53 values used; Mullin’s book [92] - 51 values used; Raevsky *et al*. tabulation [29] - 32 values used.

#### Single-source measurement of many compounds (‘quick catches’)

The small single-source databases below consist largely of intrinsic solubility values. Useful collections of original measurements included those of McFarland *et al.* [93], Bergström and coworkers [94-98], and Faller and Ertl [77].

- Avdeef [80] - 39 values, not published elsewhere, were used.
- Rytting *et al.* [99] - free-base/acid (no salts used) SSF-measured *S*_w_: solubility of 113 molecules, all measured in one laboratory, with *S*_0_ calculated by *p*DISOL-X.
- CheqSol log *S*_0_ at 25 °C (potentiometric) - 233 values for 145 molecules collected from several publications: Stuart and Box [83], Sköld *et al.* [100], Llinàs *et al.* [19, 101], Box and Comer [102], Hopfinger *et al.* [103], Narasimham *et al.* [104], Hsieh *et al.* [105], Comer *et al.* [106], Palmer and Mitchell [74], Etherson *et al.* [107]; Schönherr *et al.* [108]; Fornells *et al*. [109], and Baek *et al.* [110].
- *p*SOL log *S*_0_ at 25 °C (potentiometric) – 75 published values were collected: Avdeef [111, 112], Avdeef *et al.* [82], Avdeef and Berger [113], Faller and Wohnsland [114], Bergström *et al.* [115], Fioritto *et al.* [116], and Ottaviani *et al*. [117].

#### Data from miscellaneous primary sources (‘deep-sea fishing’)

About 2000 solubility values were gathered from various primary (non-database) sources. Those publications which contained measurements as a function of pH were particularly valuable. A large fraction of the primary source data originated from a few journals: *Int. J. Pharm.*, *J. Pharm. Sci.*, *Pharm. Res.*, *J. Chem. Eng. Data*, *Eur. J. Pharm. Sci*., *AAPS PharmSciTech, AAPS J, J. Chem. Inf. Comput. Sci.,* and *Ind. Eng. Chem. Res*.

#### Sources of p*K*_a_ data

The p*K*_a_ values of the ionizable molecules were taken from Avdeef [80]; (*cf*., www.in-ADME.com/wiki_pka.php/), and various other established sources. When no experimental values were found, then the values calculated by MarvinSketch 5.3.7 (ChemAxon Ltd., www.chemaxon.com) were used. The p*K*_a_ values were automatically adjusted for changes in the ionic strength [11, 80] and temperature [118] by *p*DISOL-X.

#### Units conversion

Solubility data have been reported in many concentration units: mol/L (molarity, M), mM, μM, mol/kg (molality, m), mole fraction (x), mg/mL, μg/mL, mg/100mL, mg/dL, %w/v, g%mL, mg/mL%, mg%, “1 in 15 of water,” “soluble in 3 parts of water,” “2% soluble in water,” units of IU/mL, etc. Mole fraction and molality units are almost always used when solubility is determined over a wide range of temperatures, since the units do not depend on the density of the solutions. In the clearly presented accounts, the *equivalent* molecular weight to use to convert the practical units (*e.g.*, μg/mL) to molarity is stated (*e.g.*, “concentration is expressed as *free base equivalent*”). In practice, it is *all too easy to make a mistake* in converting the reported units to the preferred molarity scale, so extra care is recommended.

It could be argued that solubility should be tabulated in logarithmic units (preferably based on molarity). (i) Direct values span over 12 orders of magnitude and cannot be accurately depicted in *S*-pH plots at the low end of the scale (*sic* - log of “zero” solubility is undefined). Unfortunately, raw *S*-pH data are often presented *only* in a plot, with points plotted at ~zero. (ii) Errors in log *S* values do not depend on the magnitude of the log *S* (whereas they do when direct units are considered). This is problematic when refinement of constants is based on *S* measurements and unit weights are assumed.

In the *Wiki-pS_0_* database, values reported in molality units are noted, but are seldom converted to those in molarity (by applying solution density), since the differences are small around the temperature range of interest, and since solution density is usually not reported.

#### Interlaboratory reproducibility

There are 870 different molecules in *Wiki-pS_0_* for which solubility was reported from at least two different sources. This formed the basis for estimating interlaboratory reproducibility. Some molecules had been studied in many different laboratories. For example, there were 33 different reports of the solubility of diclofenac found to date, with 17 of these measured at several different pH values, whose complicated profiles were reconciled and discussed by Bergström and Avdeef [79]. The next most-frequently studied molecules are phenytoin, barbital, and ketoprofen, with 30, 26, and 24 interlaboratory determinations, respectively. The average interlaboratory reproducibility, *SD*_avg_, based on the curated 870 replicated studies, has been determined to be 0.17 log unit, significantly lower than the experimental reproducibility suggested in past studies (~0.6 log unit) [72-74]. As noted above, many factors can lead to the perception of poor reproducibility of measurements. It takes some effort to factor in the possible sources of systematic error, to attain the low *SD*_avg_. Still, for some difficult-to-measure drug molecules, the intrinsic solubility is quite uncertain, with *SD* values exceeding 0.5 log unit [20, 79].

### Physicochemical properties of database molecules

**Figure 2.**
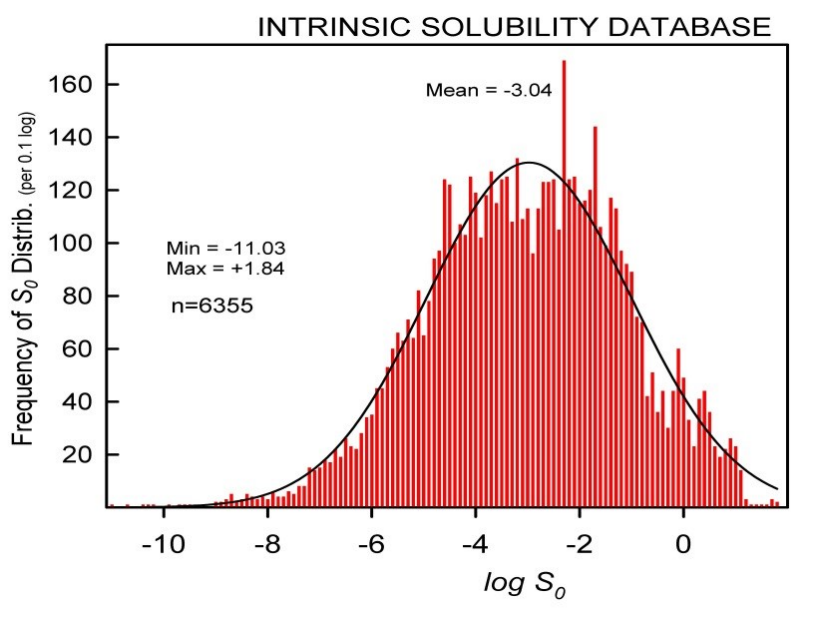
Distribution of intrinsic solubility values in *Wiki-pS_0_.*

The 6355 intrinsic solubility set ranges in log *S*_0_ from -11.0 to +1.8 (log molarity), essentially with a Gaussian distribution: mean = -3.04, median = -3.00, *SD* = 1.88. Figure 2 shows the solubility distribution for the molecules. About 47% of the entries have log *S*_0_ between -7 and -3, the typical range (DOA – domain of applicability) of values for drugs and research compounds [65]. About 2% of the molecules have log *S*_0_ < -7. Some of the least-soluble molecules (log *S*_0_ < -8) in the database are amiodarone < clofazimine < itraconazole < halofantrine < ubiquinone < epristeride < vinorelbine < silafluofen < cosalane < etretinate < probucol < arotinoic acid < clomifene < motretinide < lasalocid < carbenoxolone. The most soluble (log *S*_0_ > 0) substances are amino acids, simple carboxylic acids, and carbohydrates.

**Figure 3.**
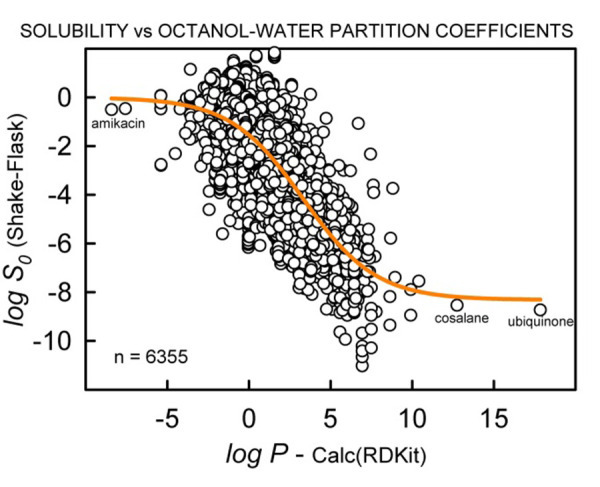
Plot of log *S*_0_ (largely SSF type) versus octanol-water partition coefficient, log *P*, calculated using the RDKit software [15].

Figure 3 shows the trend between measured log *S*_0_ and calculated log *P* (RDKit [15]), the most important descriptor in the prediction of solubility. The scatter is substantial, and perhaps trends nonlinearly at the extremes of the scales. The measured extreme values of log *S_0_* are possibly more accurate (since these are mostly determined from multi-point log *S*-H profiles) than the corresponding calculated log *P* (*cf*., ubiquinone and amikacin log *P* values). The traditional shake-flask method for direct-measure log *P* is thought to be limited to the range (-2 to +5), so methods for prediction of log *P* would be hard pressed to extrapolate accurately beyond that range, in the absence of reliably measured log *P* training-set values.

**Figure 4.**
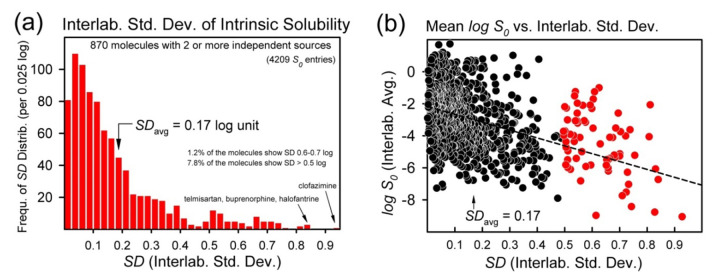
Interlaboratory reproducibility, as indicated by *SD*, was determined from averaging log *S*_0_ derived from different sources. (a) Error distribution for the 870 replicates. (b) Interlaboratory average log *S*_0_ plotted against the corresponding *SD* values. The trend suggests that the lowest solubility values have the highest errors, but the data scatter is high.

Figure 4 shows the distribution of errors determined by averaging the log *S_0_* of those replicate molecules measured in different laboratories. The average value of interlaboratory standard deviation is *SD_avg_* = 0.17 log unit. The individual *SD* values trend to higher values as solubility decreases (Fig. 4b).

The molecule showing the poorest reproducibility, with *SD* = 0.93 log unit (avg. from five sources), is clofazimine. It is also among the least soluble molecules in the database, with average log *S*_0_ *=* -9.05. The weakly dibasic (p*K*_a_ 3.83, 7.54 at 37 °C, *I* = 0.15 M [105]) phenazine antibiotic (*M*_W_ 473.4 g/mol) is used to treat leprosy. The orally-bioavailable molecule has the very unusual characteristic of precipitating and accumulating as easily-visible red microcrystals in macrophages [119].

### Rule of 5 characteristics

**Figure 5.**
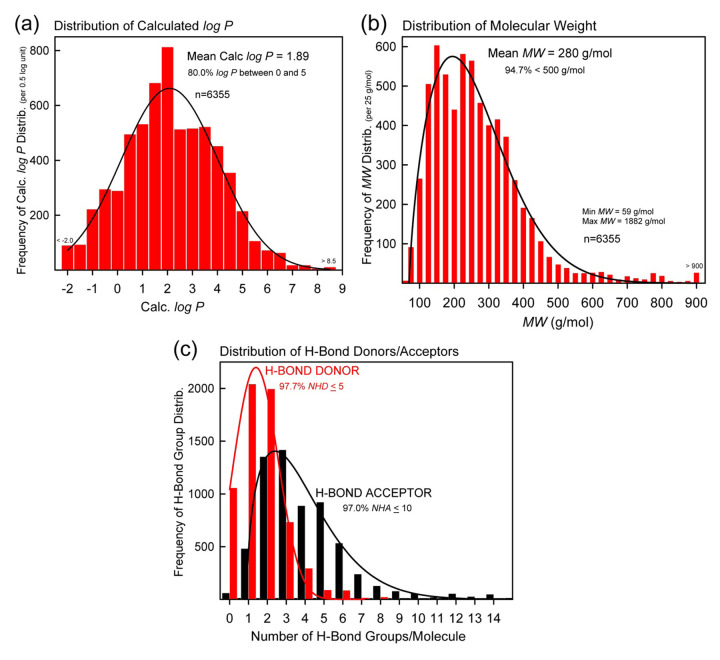
Rule of 5 property distributions: **(a)** log *P*, **(b)** molecular weight (*M*_W_), and **(c)** number of H-bond donors (*NHD*) and acceptors (*NHA*). Most of the molecules have ‘druglike’ properties.

Figure 5 shows the distribution of properties used by Lipinski *et al.* [120] to define the Rule of 5 as an indicator of “drug-likeness.” Frame (a) shows the log *P* distribution, with the average value of 1.89. About 80% of the 6355 entries fall within the range of 0 to 5 (expected range for druglike molecules). Frame (b) shows the distribution of molecular weights, with the mean value 280 g/mol. About 95% of the molecules have *M*_W_ < 500 g/mol (‘good’ range). Frame (c) considers H-bonding characteristics. The red bars (tallest) refer to H-bond donor counts (*NHD*), where 98% *NHD* ≤ 5 (‘good’). The black bars (extending to higher counts) refer to H-bond acceptors (*NHA*), where 97% *NHA* ≤ 10 (‘good’). For the most part, the database molecules are in the expected boundaries of drug-likeness, with log *P* showing some violations at the high end, and more so at the low end for about 20% of the entries.

## Results and discussion

Table 1 summarizes the results of the weighted multiple linear regression (MLR) analysis of the Abraham solvation equation (ABSOLV), and the ‘trained’ version of Yalkowsky’s general solubility equation (GSE). Also listed are the Random Forest regression (RFR) metrics. The 22 quaternary ammonium compounds were treated as a separate subset, using just some of the Abraham descriptors. The remaining 6333 solubility values were subjected to the full MLR analyses. Furthermore, the molecules were considered separately in each of four acid-base classes – with reference to predominant charge state at pH 7.4: acids(-), bases(+), neutrals(0), and zwitterions(±), as well as in combined classes.

### Yalkowsky’s general solubility equation (GSE)

**Figure 6.**
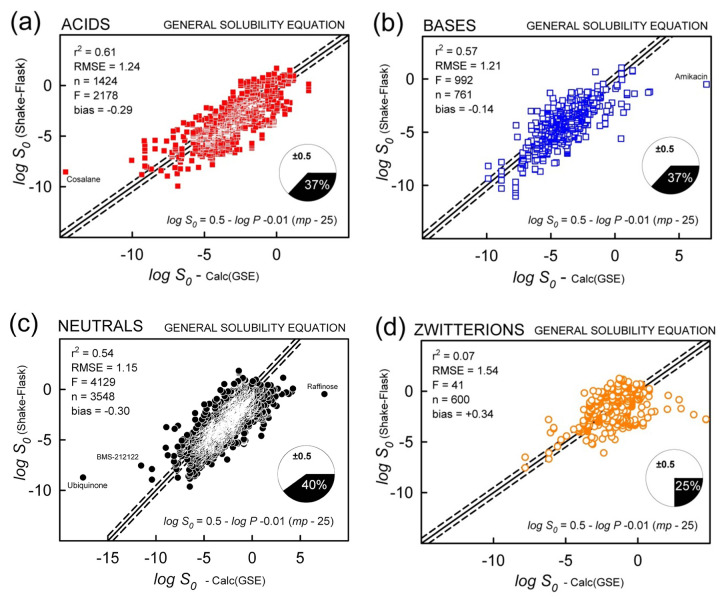
Prediction of the *Wiki-pS_0_* database log *S_0_* values using Yalkowsky’s General Solubility Equation (GSE), Eq. 1. The molecules are divided into four acid-base classes with reference to predominant charge state at pH 7.4: acids(-), bases(+), neutrals(0), and zwitterions(±). The solid diagonal is the identity line. The dashed lines are displaced from the identity line by ±0.5 log. The pie chart refers to the percentage of ‘correct’ predictions, MPP (measure of prediction performance).

It was of interest to see how well the GSE (untrained) predicted solubility values in the database. Figure 6 shows the results of applying Eq. 1 to the acid-base subset data. The first three classes (Figs. 6a-c) have similar statistical metrics: r^2^ = 0.54 to 0.61, RMSE = 1.15 to 1.24, bias = -0.14 to -0.30, and MPP = 37-40% (measure of prediction performance: percentage of the absolute residuals ≤ ±0.5 log unit). The GSE did not perform as well for the zwitterions (Fig. 6d): r^2^ = 0.07, RMSE = 1.54, bias = +0.34, and MPP =25%. The average calculated log *P* [15] for the zwitterion set is 0.07 (Table 1), suggesting that the GSE prediction of zwitterions is based largely on *T*_m_ contributions. When all the classes were combined (n = 6333, excluding 22 quaternary ammonium drugs), the untrained GSE prediction yielded r^2^ = 0.57 and RMSE = 1.23 (Table 1).

When the fixed coefficients in Eq. 1 (0.5, -1.0, -0.01) were subjected to regression using weighted MLR, the fit improved only slightly for the combined acid-base classes: r^2^ = 0.60, RMSE = 1.17, n = 6333, but the refined coefficients (-0.33, -0.83, - 0.006) were quite different from the traditional values, especially for the intercept coefficient (Table 1). This may be due to the negative correlation between the intercept and the *T*_m_ terms (-82 to -97%). When the molecules were examined by the acid-base classes, the *acids* most resembled the results of the untrained GSE, with coefficients (0.62, -0.94, -0.0115) and metrics: r^2^ = 0.70, RMSE = 1.02, n = 1424. The bases and neutrals indicated a negative intercept, -0.28, with only slightly improved metrics (Table 1). The zwitterion class had reversal of signs for both the intercept and the temperature dependence coefficients, with the slightly improved metrics: r^2^ = 0.22, RMSE = 1.28, n = 600.

### Weighted multiple linear regression using Abraham descriptors (ABSOLV)

**Figure 7.**
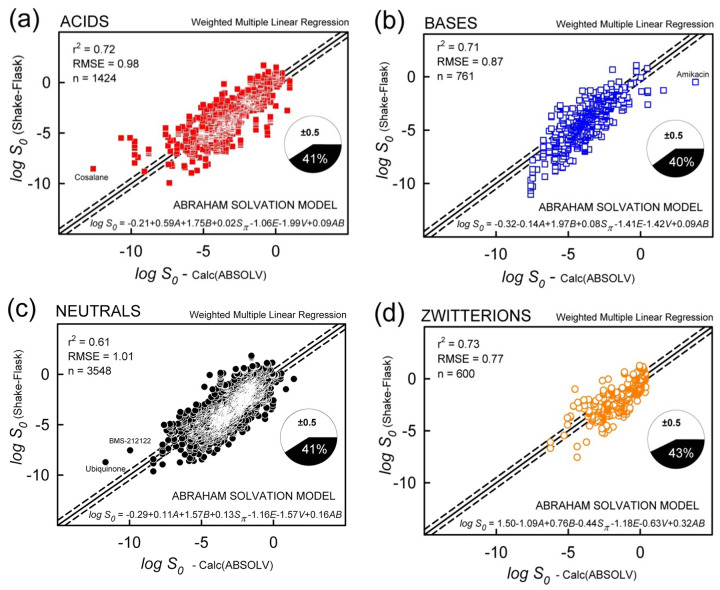
MLR prediction of log *S*_0_ in the *Wiki-pS_0_* database using Abraham Solvation Equation (ABOLV), Eq. 2. The molecules are divided into four acid-base classes with reference to predominant charge state at pH 7.4: acids(-), bases(+), neutrals(0), and zwitterions(±). The solid diagonal is the identity line. The dashed lines are displaced from the identity line by ±0.5 log. The pie chart refers to the percentage of ‘correct’ predictions, MPP (measure of prediction performance).

Figure 7 displays, by acid-base classes, the results of the weighted MLR analysis using the five Abraham ABSOLV descriptors plus the *A·B* cross-product term. The statistical metrics were similar for the four classes: r^2^ = 0.61 to 0.73, RMSE = 0.77 (zwitterions) to 1.01 (neutrals), and 40-43% ‘correct’ values (MPP). The performance was slightly better than that of the GSE (trained or untrained), and a lot better in the case of zwitterions. The refined ABSOLV coefficients (Table 1) indicate acid-base class differences. These coefficients are not similar to the ones reported by Abraham and Le [13]. In MLR, such differences in coefficients can arise when different training sets are used, as a result of correlations between descriptors. It was found that *const:A* correlations ranged -50 to -83% and *const:AB* correlations ranged +57 to +79%.

### Random Forest regression using RDKit combined with Abraham descriptors and melting points

#### Descriptors

For the RFR model building, the 193 RDKit (2014 version) descriptors calculated were pooled with the *T*_m_ (81% values measured, the rest calculated) and the calculated ABSOLV descriptors. The *Abbreviations and definitions* section below identifies and defines the most important descriptors used in the RFR algorithm.

#### Training set and internal validation

**Figure 8.**
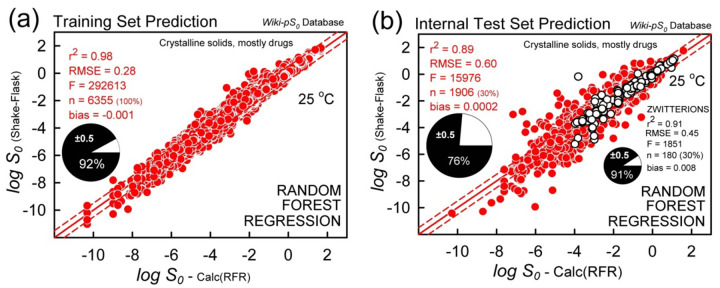
Random Forest regression analysis. The solid diagonals are the identity lines, and the dashed lines refer to ±0.5 log deviations. The MPP pie charts refer to percentage of ‘correct’ prediction, with absolute residuals ≤0.5 log. (**a**) Training set using the entire database. (**b**) Internal test sets, based on 30% of the database. The unfilled-circle symbols correspond to the zwitterion internal test set (30% of 600).

Figure 8a shows the entire training set RFR analysis, with the metrics: r^2^ = 0.95, RMSE = 0.40,  bias = -0.007. This is *not* a good measure of the predictive power of the method. Rather, it indicates how well the model can incorporate the information represented by the descriptors and relate it to solubility in the training set [66]. The randomly selected internal test set of 1906 solubility values (30%) are better indicators of the ability of the model to predict external tests compounds which are unknown to the training process. Figure 8b shows the *internal* test set prediction results: r^2^ = 0.89, RMSE = 0.60,  bias = 0.0002. This performance is to be expected for *external* test molecules which are well-represented by the chemical space of the database, as illustrated below.

The bottom section of Table 1 summarizes the analysis metrics, both for the entire data set and for the acid-base subsets. The best internal test set performance was found for the zwitterions: r^2^ = 0.91, RMSE = 0.45. The right-most column identifies the ten most-important descriptors in the analysis. For the overall data, and for the acid, base, and neutral subsets, the most important descriptor is log *P*. It’s particularly noteworthy that log *P* is not in the top-10 list for the zwitterions. In several of the cases, the second-most important descriptor is molecular refractivity (cf., *Abbreviations and definitions* for the RDKit terminology). Topological indices play particularly important roles in the zwitterion subset.

#### Principal component analysis of thirty of the most important RDKit descriptors

**Figure 9.**
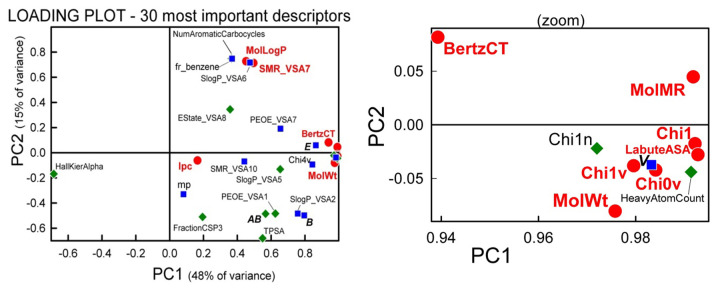
Principal components analysis loading plot for the 30-most important RFR descriptors. The zoom view identifies highly-correlated size-related descriptors. Circles represent the 10-most important descriptors; squares represent the second 10-most important descriptors; diamonds represent the remaining ranking.

The principal component analysis (PCA) function, prcomp(), in the factoextra R library was used to process the 30-most important descriptors identified in RFR. Figure 9 shows the loading plot based on the first two principal components, which account for 63% of the total variance in the descriptors. Only the HallKierAlpha descriptor has a negative PC1 value, with all of the rest of the descriptors being in the positive PC1 domain. The close proximity of many of the descriptors to each other suggests high correlation between them. Such correlations would be problematic in MLR analysis, but not in RFR.

**Figure 10.**
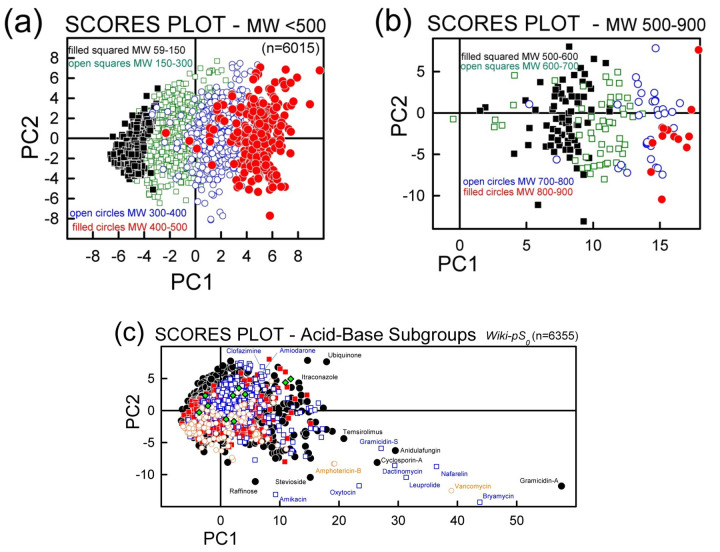
Principal components scores plot for the RFR training set. (a) Molecular weights < 500 g/mol; (b) MW > 500 g/mol; (c) “Comet-shaped” distribution for the entire database by acid-base classes. Symbols have the same meaning as in Figures 6 and 7. The green diamonds refer to quaternary ammonium drugs.

Figure 10 shows the scores plots for the solubility data. Frame (a), which considers only the molecules with *M*_W_ < 500 g/mol, shows a very dense but apparently symmetrical distribution about the origin. As *M*_W_s increase, the points shift in the direction of increasing PC1. Frame (b) shows the molecules with MW > 500 g/mol. The distribution is sparse and further shifted to increasing PC1 values, as *M*_W_ values increase. Frame (c) shows all the data with the acid-base subset notation. Very large molecules are thinly represented in the bottom-right quadrant. Zwitterions tent to be in the negative PC2 half, evenly distributed in PC1.

#### Validation against four external test sets

Four external test sets were selected to explore how well the GSE, ABSOLV, and RFR models perform. For each of the test sets, *all the test molecules found in the training set were removed*, so that the prediction was of truly “unknown” molecules. This was not necessary for the traditional GSE model, since it requires no training. The observed and calculated values are listed in Appendix Tables A1-A4.

**Figure 11.**
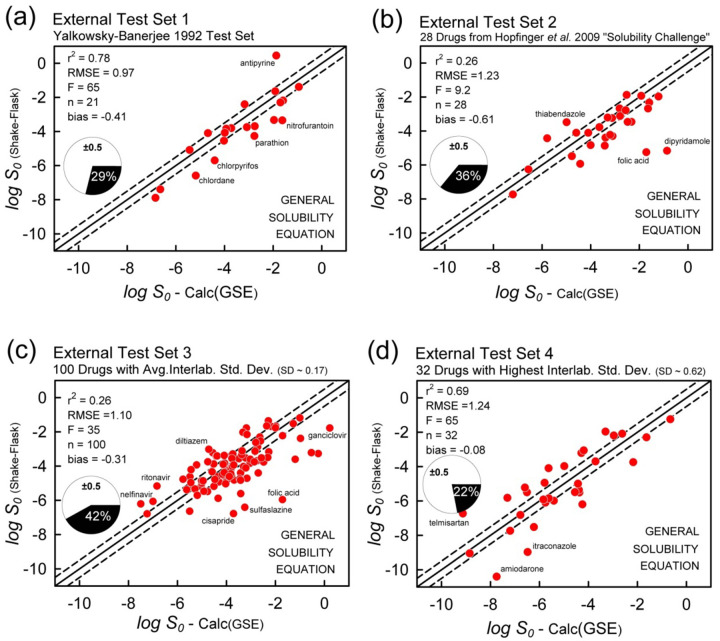
GSE (“untrained”) prediction (Eq. 1) of the four external test sets. RDKit log *P* was used.

Figure 11 displays the correlation plots of the GSE calculation for each of the four test sets, using RDKit-calculated log *P*. RMSE range from 0.97 to 1.24, as 22 – 42% of the data are ‘correctly’ predicted (MPP).

**Figure 12.**
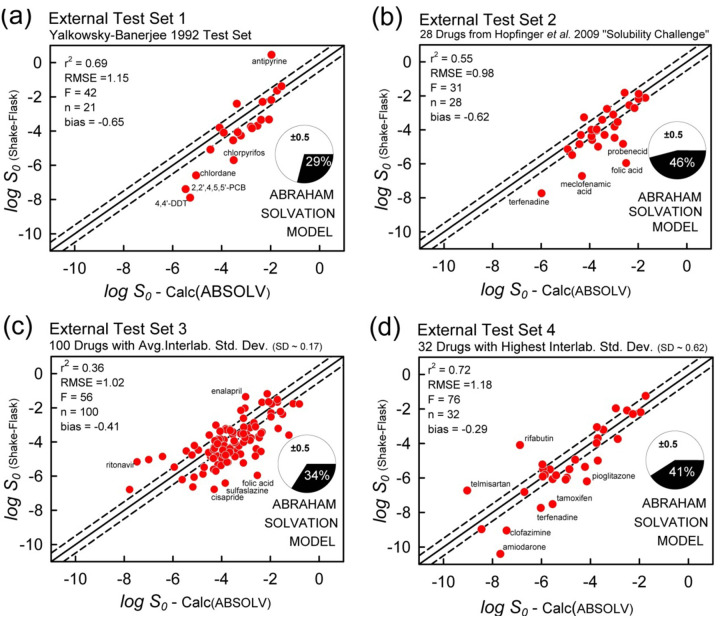
ABSOLV weighted MLR prediction (Eq. 2) of the four external test sets. The Abraham Solvation Equation was trained with the druglike *Wiki-pS_0_* database.

Figure 12 displays the correlation plots of the ABSOLV weighted MLR analysis for each of the four test sets. The ABSOLV model predicted the Hopfinger *et al.* Test Set 2 better than did the SGE model (RMSE 0.98 vs. 1.23), but did not do as well with Test Set 1 (RMSE 1.15 vs 0.97). The performances with Test Sets 3 and 4 were comparable between GSE and ABSOLV models, with RMSE values ranging from 1.02 to 1.24.

**Figure 13.**
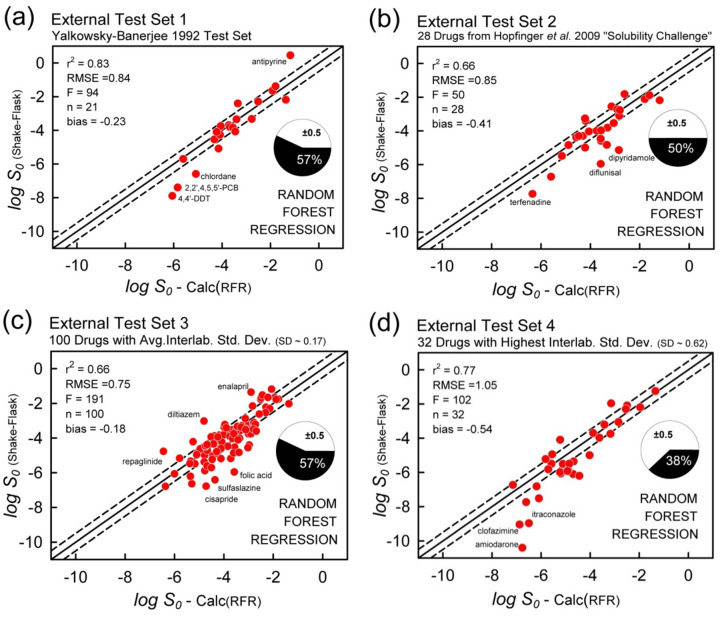
RFR prediction of the four external test sets. With 3 outliers removed (n=29) in (d), r^2^ = 0.82, RMSE=0.76, F=121, bias = -0.31, with 41% residuals falling inside the dashed lines.

Figure 13 displays the correlation plots of the RFR model for each of the four test sets. The overall statistics (r^2^ = 0.66-0.83, RMSE = 0.75-1.05) indicate that the predictions are better than those in the other two models.

However, there were two main problem areas in the RFR modeling, as indicated by poor fit: (i) Fig. 13a shows the outlier pesticides 4,4’-DDT, 2,2’,4,5,5’-PCB and chlordane; (ii) Fig. 13d shows the outlier drugs amiodarone, clofazimine, and itraconazole.

**Figure 14.**
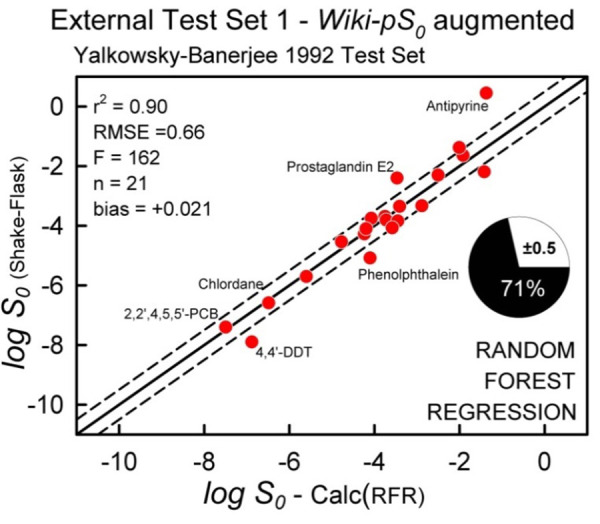
Prediction of Test Set 1 molecules with an *augmented* RFR training set.

Case (i) can be remedied. The *Wiki-pS_0_* database has very few agrochemicals and no DDT or PCB derivatives. We decided to temporarily augment our database with agrochemicals, to see if RFR prediction could be improved for Test Set 1 (Fig. 13a). The Huuskonen [35] database of 1297 organic molecules was screened with three filters: (a) only compounds with log *S_w_* < -5 would be used; (b) only solids would be considered; and (c) Test Set 1 compounds would be excluded. This process resulted in 115 new entries to the augmented database. Figure 14 shows the improved results. By adding a few agrochemicals to the RFR training set, r^2^ increased from 0.83 to 0.90, RMSE decreased from 0.83 to 0.66, bias lowered from -0.23 to +0.02, and ‘correct’ predictions increased from 57 to 71%. The well-known adage that “like predicts like” is amply illustrated in this example.

Antipyrine appears to be poorly fit for reasons related to uncertainty in calculated log *P* (calc: 1.48, obs: 0.38). Replacement of the calculated with the observed value improved the antipyrine fit by 0.2 log units, suggesting that other descriptors may be problematic. (The improvement in the GSE calculation was 1.2 log units for antipyrine.)

Case (ii) remains problematic - a case of training-set “missing neighbors” problem. As is evident in Fig. 13d, amiodarone, clofazimine, and itraconazole are poorly predicted, in part because there are few other molecules possessing the properties of these three compounds (cf., upper right edge in scores plot Fig.10c) in the database, and also, because RFR cannot extrapolate solubility beyond the range of its training data. From the PCA analysis, the five nearest neighbors to amiodarone (log *S*_0_ = -10.4), based on three principal components, are halofantrine, irbesartan, butaperazine, mifepristone, and probucol. The log *S_0_* values for these neighbors show high variance: -8.0, -3.7, -4.3, -5.2, and -8.4, respectively. The RFR-predicted value for amiodarone is log *S*_0_ = -6.8, barely greater than the average value of the five nearest neighbors. To do better, the database needs new neighbors in the chemical space close to amiodarone, clofazimine, and itraconazole. Or, better descriptors are needed to define the chemical space, so that truly ‘similar’ molecules will have nearly the same solubility values. With the three outliers removed, the metric improve: r^2^ = 0.82, RMSE = 0.76, bias = -0.31, and MPP = 41%.

### Prediction of solubility of quaternary ammonium drugs

**Figure 15.**
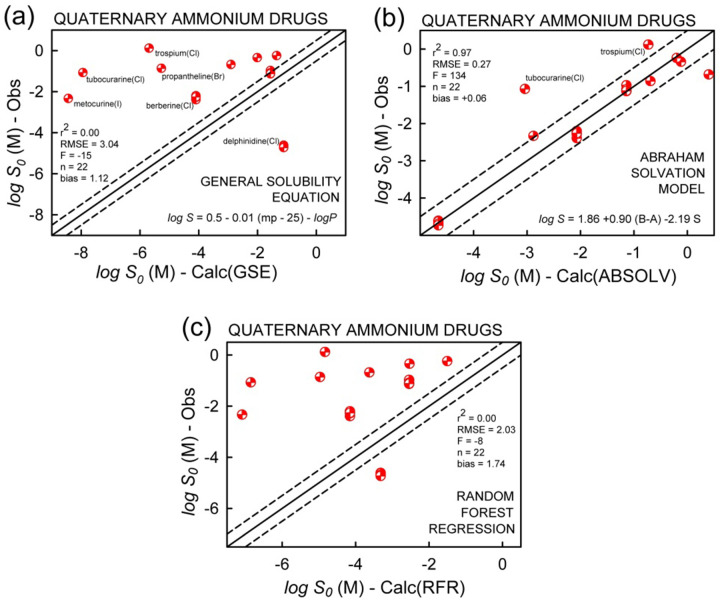
Prediction models for quaternary ammonium compounds. Here, *S*_0_ represents the quaternary ammonium salt solubility, *S*_QA_.

Quaternary ammonium compounds are salts, and so do not fall into the category of neutral species associated with the log *S_0_* constants studied here. GSE and RFR methods did not produce satisfactory results (r^2^ ~ 0 in both cases) for this subclass of compounds. However, it was possible to come up with a modified ABSOLV model for this small group of molecules (n=22), based on the equation:


(4)

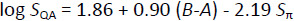



with r^2^ = 0.97 and RMSE = 0.27, where *S*_π_ in Eq. (4) is the dipolarity/polarizability Abraham descriptor. Figure 15 compares the tested calculations. Strong H-bond donors (acids) decrease solubility, whereas strong H-bond acceptors (bases) have the opposite effect. High dipolarity/polarizability compounds are associate with low solubility.

## Summary

The properties of the chemical space of druglike molecules in the *Wiki-pS_0_* database of intrinsic aqueous solubility were described in considerable detail. The database was used to train two solubility prediction models: multiple linear regression (MLR) and Random Forest regression (RFR). The predictivity of the models was tested with four external sets of compounds. The MLR model incorporated calculated Abraham solvation descriptors (ABSOLV). The RFR model used the aggregate set of *T*_m_ (mostly measured values), ABSOLV, and RDKit 2D (204 descriptors in all). As a comparative benchmark, the General Solubility Equation (GSE), which requires no training, was used to predict the intrinsic solubility of the *Wiki-pS_0_* druglike molecules.

For the intrinsic solubility set, excluding the permanently-charged quaternary amines, RMSE calculated as 1.23 (GSE), 1.00 (ABSOLV), and 0.28 (RFR) for the training sets. The intrinsic set was further divided into four subsets, based on dominant charge at pH 7.4: acids(-), bases(+), neutrals(0), and zwitterions(±). The performances of GSE and ABSOLV were comparable for acids, bases, and neutrals, but for the zwitterionic subset, ABSOLV was better.

For the permanently-charged quaternary amines (n=22), both GSE and RFR did not do well (r^2^ = 0). It was possible to develop a simplified ABSOLV training-set model using just three of the solvation descriptors.

The above comparisons are not entirely satisfactory tests of the *predictivity* of the three methods. For the RFR method, the data are randomly separated into a training set (70%) and an internal test set (30%). RMSE = 0.60 and MPP = 76% ‘correct’ predictions for the internal test set calculation. For the zwitterionic subset, RMSE = 0.45 and MPP = 91%.

The four *external* test sets allowed the comparisons of the three models in a uniform way. Test Set 1 (te1) was compiled by Yalkowsky and Banerjee [18] for testing the GSE. The other three test sets consisted of druglike molecules, all solids at room temperature, containing no agrochemicals. Test Set 2 (te2) molecules were originally used in the first Solubility Challenge [19, 103], and Test Sets 3 (te3) and 4 (te4) molecules were used in the second Solubility Challenge [20].

The GSE applied to simple organic compounds (te1) indicated RMSE = 0.97 and MPP = 29% ‘correct’ predictions. When *experimental* log *P* values are used in Eq. (1) [18], the te1 performance improves: RMSE = 0.72 and MPP = 52%.

RFR outperformed the other two methods on the whole. When *Wiki-pS_0_* was augmented with 115 agrochemicals, te1 prediction improved (RMSE = 0.66, MPP = 71%), and was better than that of GSE. For te2 and te3 drug solubility RFR predictions, RMSE = 0.85 and 0.75, resp., whereas MPP = 50 and 57%, resp. There were three molecules in te4 that RFR did not predict well: amiodarone, clofazimine, and itraconazole. Apparently, the current database has limited chemical space coverage in the vicinity of these outliers. With the three outliers removed, RMSE = 0.76 and MPP = 41% for te4.

## Conclusion

The GSE is popular for its simplicity and easy of calculation. It is a convenient benchmark against which to assess new prediction methods. Druglike molecules are expected to be predicted by GSE to within 1.1-1.2 log unit, or to within 0.5 log unit 22-42% of the time. However, its performance with zwitterionic molecules is limited. The ABSOLV method holds the middle position in the comparisons. The RFR method in this study is attractive, both for its predictive performance and ease of use. It is expected to predict druglike molecules similar to those in *Wiki-pS_0_* to within 0.6 log unit of the measured values, or within 0.5 log unit 76% of the time. The RFR software is freely downloadable from open sources.

Evidently, the evaluated prediction methods cannot match the precision of measured equilibrium solubility data. The methods need to be further enhanced. More discriminating descriptors would be welcome additions to the openly-available collections. As the amiodarone, clofazimine, and itraconazole examples illustrate, there are still under-populated neighborhoods in the chemical space of the currently tested database. How effective *Wiki-pS_0_* will be in predicting the solubility of newly-synthesized molecules in pharmaceutical research remains to be explored.

## Abbreviations and definitions

**Table:** 

DOA	domain of applicability associated with druglike substances, determined by descriptor or structural (*e.g*., Tanimoto indices) similarity.
DTT	Dissolution Titration Template potentiometric method used to determine intrinsic solubility, S_0_
HH	Henderson-Hasselbalch equation [80]; *e.g.*, for monoprotic base, log *S* = log *S*_0_ + log ( 10 ^+p*K*a – pH^ + 1 )
OOB	“Out-of-Bag” built-in validation set of compounds randomly selected by the RFR method, which have not been used to train the model.
pH_sat_	the equilibrium pH of a saturated water solution of compound whose solubility is S_w_
*S*	solubility, ideally expressed in units of mol/L (M), μg/mL, or mg/mL
*S*_0_	“intrinsic” solubility (i.e., the solubility of the *uncharged* form of the compound)
*S*_w_	“water” solubility, defined by dissolving enough pure free acid/base in distilled water (or water containing an inert salt - as ionic strength adjustor) to form a saturated solution. The final pH of the suspension, pH_sat_, and *S*_0_ can be calculated by the HH equation (when valid), provided the true p*K*_a_ is known. Compound added in salt form may disproportionate into free acid/base, depending on how much solid had been added. Calculation of the pH and S_0_ of such salt suspensions can be uncertain.
*S*_pH_	“pH buffer” solubility (i.e., the total solubility of the compound at a *measured* equilibrated pH)
SSF	saturation shake-flask method, the “gold standard” solubility measurement method
RMSE	root-mean-square error: RMSE = [ 1/n Σ_i_ (y_i_^obs^ - y_i_^calc^)^2^ ]^1/2^, where y^obs^/ y^calc^ = observed/calculated value of log *S_0_* according to model, n = number of measurements of log *S*_0_
r^2^	squared linear correlation coefficient, r^2^ = 1 - Σ_i_ (y_i_^obs^ - y_i_^calc^)^2^ / Σ_i_ (y_i_^obs^ - <y>)^2^ , where y = log *S_0_*, and <y> is the mean value of log *S_0_*
*SD*	standard deviation: *SD* = [ 1/n Σ_i_ (y_i_^obs^ - <y>)^2^ ]^1/2^, where n = number of measurements, <y> = mean value of log *S_0_*
F	F-statistic: F = (n-p-1)/p · Σ_i_ (y_i_^obs^ - <y>)^2^ / Σ_i_ (y_i_^obs^ - y_i_^calc^)^2^, where p = number of regression parameters
MPP	Measure of prediction performance [103]. It refers to the percent of ‘correct’ predictions, as defined by the count of absolute residuals |log *S*_0_^obs^ – log *S*_0_^calc^| ≤ 0.5 divided by the number of measurements. MPP is represented as a pie chart in the correlation plots.
*ntree*	number of trees specified in the Random Forest regression (RFR) – typically 500
*mtry*	number of descriptors to use in the node splitting process in RFR – typically a third of the descriptors
*nodesize*	minimum number of data points in the terminal node, beyond which no splitting takes place – typically 5 measurements

### Abraham solvation descriptors

**Table:** 

*A*	H-bond total acidity
*B*	H-bond total basicity
*S_π_*	dipolarity/polarizability due to solute-solvent interactions between bond dipoles and induced dipoles
*E*	excess molar refraction (dm^3^ mol^-1^ / 10); which models dispersion force interaction arising from π- and n-electrons of the solute
*V*	McGowan molar volume (dm^3^ mol^-1^ / 100)
*A·B*	acid-base H-bonding product descriptor used in ABSOLV solubility prediction

### Most important RDKit descriptors in RFR analysis

#### Subdivided Surface Area Molecular Descriptors [121]

**Table:** 

*LabuteVSA*	sum of atomic contributions [51] to the accessible van der Waals surface area
*MolLogP*	sum of atomic contributions to octanol/water partition coefficient, log *P*
*MolMR*	sum of atomic contributions to molar refractivity, *MR*
*SlogP_VSAk*	*s*um of accessible van der Waals surface area for those atoms with atomic contribution to log *P*; k refers to a small domain of atomic-contribution to log *P*; intended to capture *hydrophobic/lipophilic effects*
*SMR_VSAk*	sum of accessible van der Waals surface area for those atoms with atomic contribution to molar refractivity; k refers to a small domain of atomic-contribution to MR; intended to capture *molecular size & polarizability*
*PEOE_VSAk*	intended to capture *direct electrostatic interactions* in a particular range; based on iterative equalization of atomic *orbital electronegativities* [49].

#### Complexity descriptors

**Table:** 

*BertzCT*	complexity index, based on size, symmetry, branching, rings, multiple bonds, and heteroatoms characteristic of solute [50].
*Ipc*	content information of topological graph [48] - entropy of atomic distribution in solute

#### Topological and electrotopological connectivity indices

*Chi0, Chi0n, Chi0v, Chi1, Chi1n, Chi4n, Chi4v, α* – Kier-Hall topological connectivity and shape indices [52, 53, 55] – numerical representations of topology of solute calculated from graphical depiction of the molecule

*Atomic and subroup counts,* HeavyAtomCount, NumberAromaticCarbocycles, NumberAromaticRings, RingCount, fr_benzene

## Availability of the *Wiki-pS_0_* Database

The entire *Wiki-pS_0_* database is planned to be released in book form: A. Avdeef. *Intrinsic Aqueous Solubility Data for Pharmaceutical Research.* Wiley-Interscience, Hoboken, NJ (under discussion with publisher). A sampling is presented in Table A5, with citations to the original literature [122-196].

## Figures and Tables

**Table 1.**
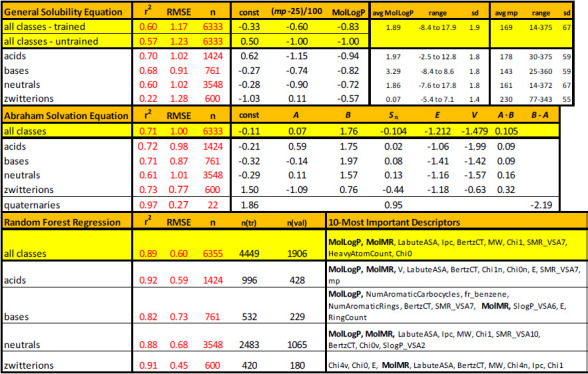
Results of log *S*_0_ prediction using three computational models^a^

^a^ Descriptors defined in Abbreviations and definitions section. n(tr) = training set count; n(val) = count for internal test set validation. The calculations with n=6333 count did not include the 22 quaternary ammonium drugs.

**Table A5.**
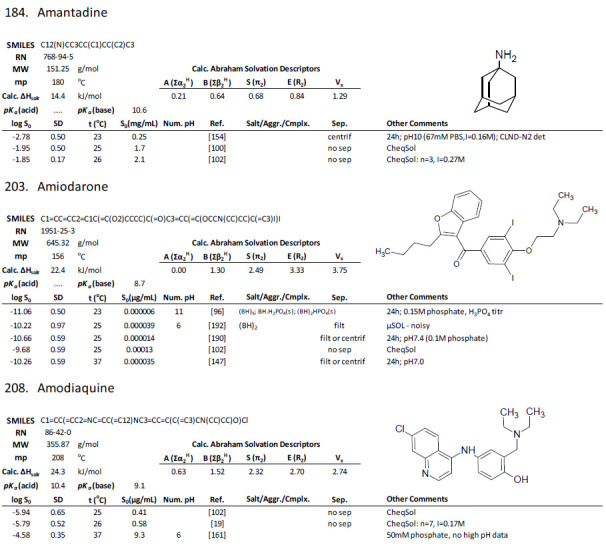

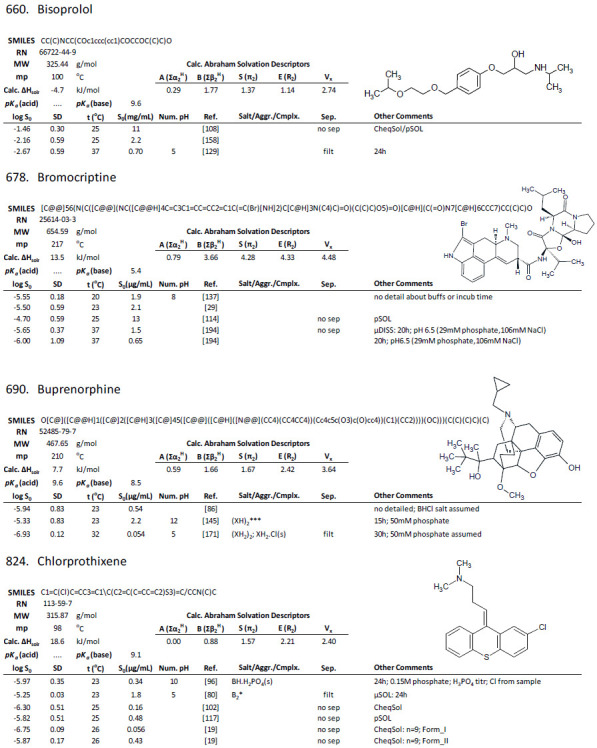

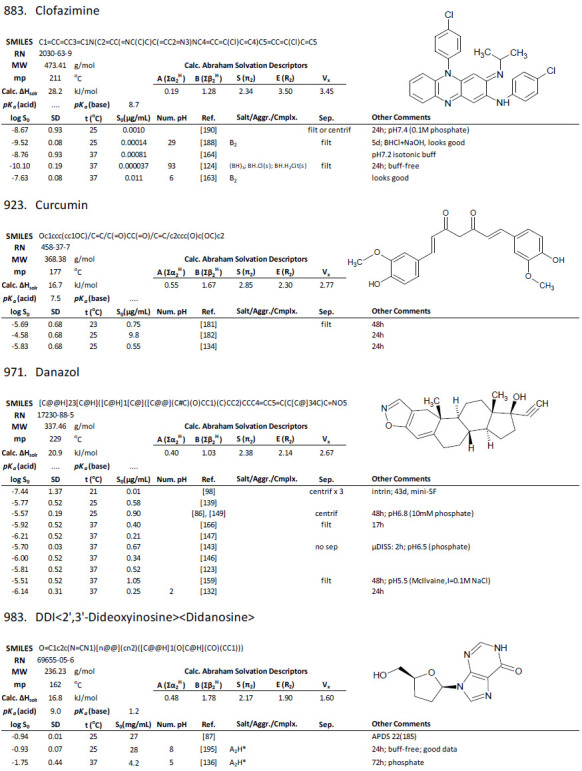

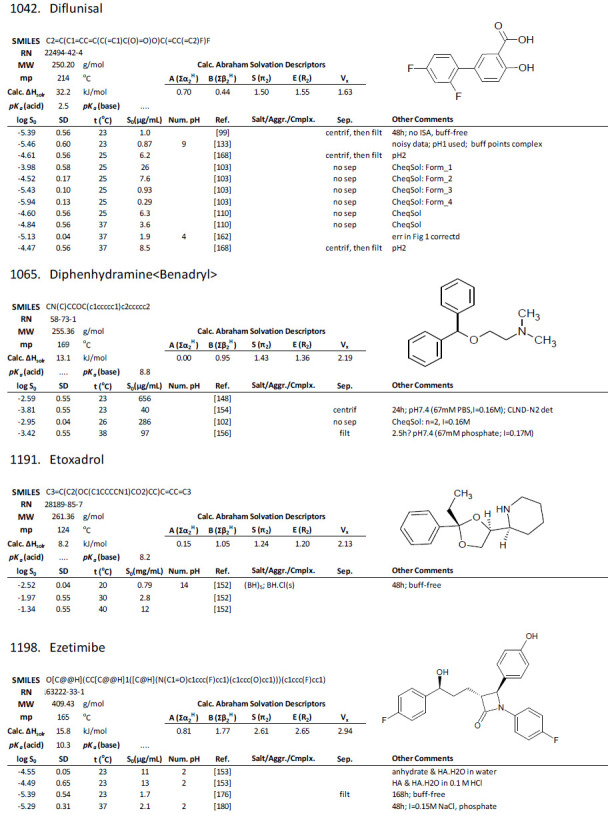

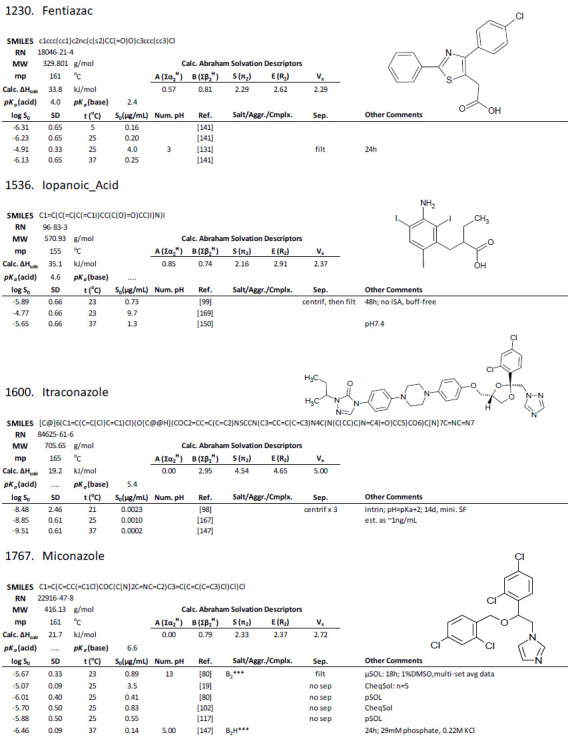

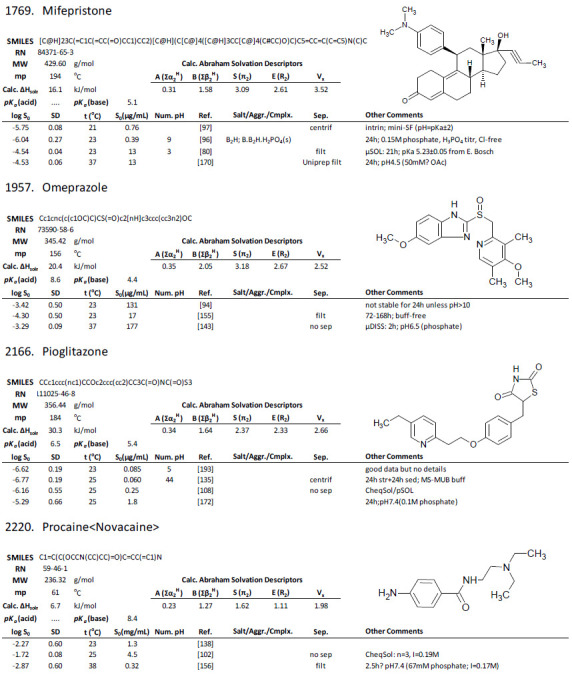

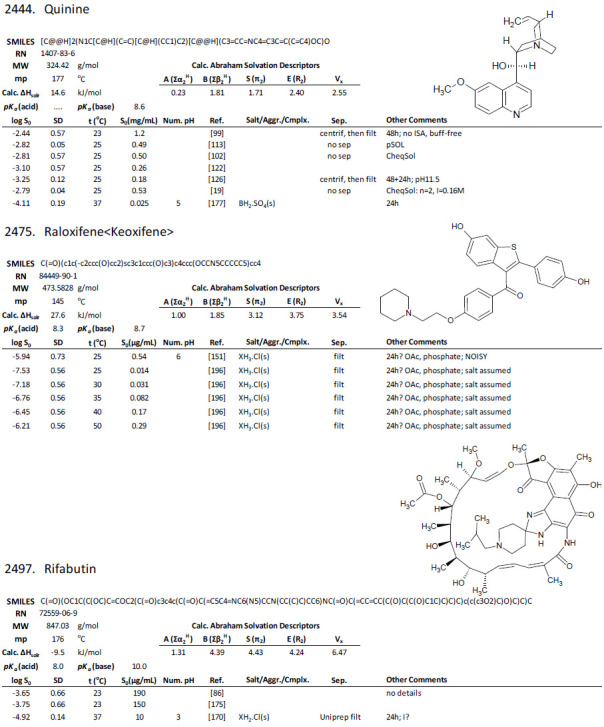

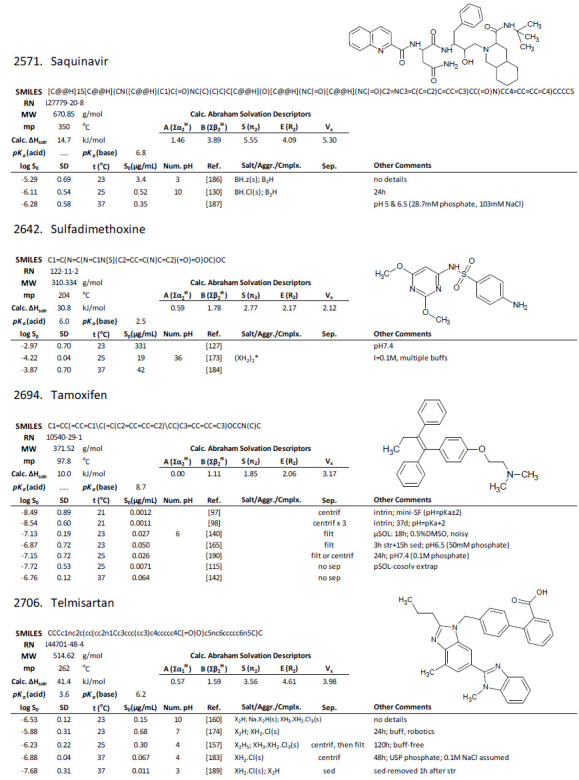

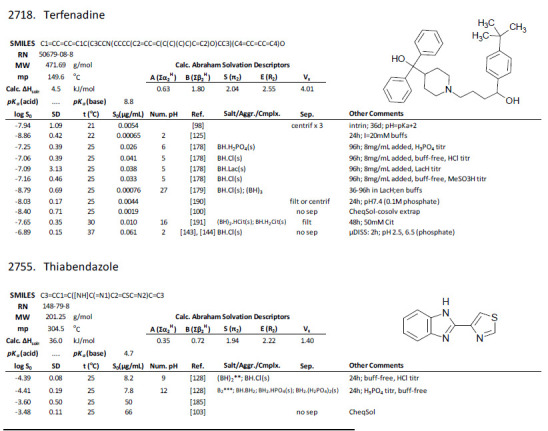
Listing of external test set four solubility values from the Wiki-pS0 database ^a^

^a^ RN – Registry number (CAS). Δ*H*_sol_ – calculated [9] heat of solubility, used to adjust data to a standard temperature (25 °C). p*K*a – calculated for strongest acid and weakest base groups. Num.pH – number of *S*_pH_ measurements in the log *S* – pH profile. *,**,*** indicate small, moderate, extensive concentration of aggregate/complex.

## Appendix – Calculated results for the three models and a sampling of the database

**Table A1.** External Test Set 1 (Yalkowsky & Banerjee,1992) ^a^

	log *S*_0_ (avg., 25 °C)			*T*_m_	log *P*	Calculated log *S*_0_		
NAME	(*Wiki-pS_0_*)	SD	n	(°C)	(RDKit)	GSE	ABSOLV	RFR
Acetylsalicylic_Acid	-1.64	0.03	28	135	1.31	-1.91	-1.74	-1.92
Antipyrine	0.45	0.08	9	114	1.48	-1.87	-1.96	-1.18
Atrazine	-3.69	0.15	6	173	1.78	-2.76	-2.54	-3.72
Benzocaine	-2.19	0.12	14	89	1.45	-1.59	-1.97	-1.36
Chlordane	-6.59	0.61	6	25	5.68	-5.18	-5.04	-5.08
Chlorpyrifos	-5.70	0.24	5	43	4.72	-4.40	-3.51	-5.61
DDT,4,4'-	-7.90	0.69	15	109	6.5	-6.84	-5.29	-6.06
Diazepam	-3.81	0.11	10	132	3.15	-3.72	-4.08	-3.68
Diazinon	-3.75	0.10	3	25	3.58	-3.08	-2.81	-4.06
Diuron	-3.84	0.09	3	159	3.09	-3.93	-2.76	-3.55
Lindane	-4.54	0.13	10	113	3.64	-4.02	-3.53	-4.32
Malathion	-3.35	0.02	9	25	2.12	-1.62	-2.39	-3.40
Nitrofurantoin	-3.33	0.11	13	264	0.07	-1.96	-2.06	-2.77
Parathion	-4.27	0.17	12	25	3.27	-2.77	-3.21	-4.08
PCB,2,2',4,5,5'-	-7.40	0.20	19	77	6.62	-6.64	-5.47	-5.84
Phenobarbital	-2.30	0.08	26	175	0.7	-1.70	-2.32	-2.51
Phenolphthalein	-5.08	0.17	2	263	3.56	-5.44	-4.46	-4.15
Phenytoin	-4.07	0.13	30	297	1.77	-3.99	-3.34	-3.45
Prostaglandin_E2	-2.40	0.09	5	67	3.25	-3.17	-3.38	-3.35
Testosterone	-4.10	0.09	16	155	3.88	-4.68	-3.91	-4.22
Theophylline	-1.38	0.09	15	273	-1.04	-0.94	-1.55	-1.79
Min.	-7.90	0.02						
Max.	0.45	0.69						
Mean	-3.85	0.17						

^a^ Melting point of liquids are set to 25 °C (chlordane, diazinon, malathion, and parathion). The measured log *P* of antipyrine is 0.38.

SD refers to standard deviation from averaging n interlaboratory reported values.

**Table A2.** External Test Set 2 (Hopfinger et al. 2009)

	log *S*_0_ (avg., 25 ^o^C)			*T*_m_	log *P*	Calculated log *S*_0_		
NAME	(*Wiki-pS_0_*)	SD	n	(°C)	(RDKit)	GSE	ABSOLV	RFR
Acebutolol	-2.56	0.31	3	119	2.37	-2.81	-2.37	-3.14
Amoxicillin	-2.12	0.07	11	194	0.02	-1.21	-1.71	-1.80
Bendroflumethiazide	-4.30	0.28	6	222	1.63	-3.10	-3.39	-4.31
Benzocaine	-2.19	0.12	14	89	1.45	-1.59	-1.99	-1.19
Benzthiazide	-4.84	0.22	6	232	2.43	-4.00	-4.42	-4.89
Clozapine	-4.60	0.12	4	184	2.03	-3.12	-3.90	-3.57
Dibucaine	-4.04	0.35	3	65	3.49	-3.39	-3.71	-4.06
Diethylstilbestrol	-4.39	0.35	7	171	4.83	-5.79	-3.92	-4.57
Diflunisal	-4.99	0.56	11	214	3.04	-4.43	-3.66	-4.21
Dipyridamole	-5.14	0.12	11	163	-0.02	-0.86	-4.91	-2.84
Folic Acid	-5.96	0.16	6	250	-0.04	-1.71	-2.50	-3.58
Furosemide	-4.47	0.22	22	206	1.89	-3.20	-2.97	-3.58
Hydrochlorothiazide	-2.72	0.10	18	274	-0.35	-1.64	-2.15	-2.91
Imipramine	-4.30	0.26	11	146	3.88	-4.59	-4.36	-4.47
Indomethacin	-5.48	0.22	21	159	3.93	-4.77	-4.72	-5.15
Ketoprofen	-3.41	0.23	24	94	3.11	-3.30	-3.48	-4.19
Lidocaine	-1.82	0.08	20	69	2.58	-2.52	-2.56	-2.62
Meclofenamic Acid	-6.72	0.31	4	257	4.74	-6.56	-4.32	-5.59
Naphthoic Acid,2-	-3.81	0.25	6	185	2.54	-3.64	-2.98	-3.30
Probenecid	-4.83	0.20	4	197	2.20	-3.42	-2.63	-3.33
Pyrimethamine	-4.00	0.47	4	233	2.52	-4.10	-3.93	-3.74
Salicylic Acid	-1.88	0.08	21	158	1.09	-1.92	-1.98	-1.61
Sulfamerazine	-3.11	0.06	7	237	1.17	-2.79	-3.03	-2.83
Sulfamethizole	-2.77	0.12	6	208	1.23	-2.56	-3.29	-2.81
Terfenadine	-7.74	0.71	11	150	6.45	-7.20	-5.98	-6.34
Thiabendazole	-3.97	0.50	4	305	2.69	-4.99	-3.71	-3.56
Tolbutamide	-3.54	0.09	7	129	1.78	-2.32	-2.85	-3.05
Trazodone	-3.27	0.20	6	87	2.36	-2.48	-4.23	-4.22
Min.	-7.74	0.06						
Max.	-1.82	0.71						
Mean	-4.03	0.24						

**Table A3.** External Test Set 3 (Avg. Interlab. SD ~0.17)

	log *S*_0_ (avg., 25 °C)			*T*_m_	log *P*	Calculated log *S*_0_		
NAME	(*Wiki-pS_0_*)	SD	n	(°C)	(RDKit)	GSE	ABSOLV	RFR
Acetazolamide	-2.38	0.18	11	259	-0.86	-0.98	-1.50	-2.29
Acetylsalicylic Acid	-1.67	0.15	16	142	1.31	-1.98	-1.71	-1.94
Alclofenac	-4.40	0.16	4	92	2.53	-2.70	-2.58	-2.97
Ambroxol	-3.87	0.17	3	234	3.19	-4.78	-3.90	-4.34
Aripiprazole	-6.64	0.21	3	139	4.86	-5.50	-5.18	-5.30
Atovaquone	-6.07	0.18	3	224	5.51	-7.00	-5.13	-6.00
Atrazine	-3.69	0.15	6	173	1.78	-2.76	-2.49	-3.83
Baclofen	-1.78	0.15	4	208	1.86	-3.19	-1.95	-2.51
Barbital,Buta-	-2.22	0.16	10	167	0.79	-1.71	-1.59	-2.30
Benzthiazide	-4.84	0.22	6	232	2.43	-4.00	-4.46	-4.65
Bromazepam	-3.39	0.13	3	193	2.63	-3.81	-3.60	-3.98
Candesartan cilexetil	-6.79	0.15	6	167	6.32	-7.24	-7.78	-6.37
Carbamazepine	-3.22	0.16	15	192	3.39	-4.56	-3.83	-3.96
Carbazole	-5.19	0.19	3	246	3.32	-5.03	-3.74	-4.12
Carbendazim	-4.56	0.19	4	320	1.74	-4.19	-2.39	-3.03
Cefmenoxime	-3.27	0.14	7	187	-0.87	-0.25	-3.66	-2.84
Cefprozil	-1.68	0.20	4	222	0.71	-2.18	-2.35	-2.49
Celecoxib	-5.89	0.18	6	158	3.51	-4.34	-4.77	-4.77
Cephradine	-1.18	0.13	8	140	0.35	-1.00	-2.13	-2.07
Chlorpropamide	-3.17	0.14	7	128	1.74	-2.27	-2.83	-3.11
Cholic Acid, Deoxy-	-4.62	0.15	7	176	4.48	-5.49	-4.44	-4.74
Cilostazol	-4.93	0.13	3	160	3.46	-4.31	-4.35	-4.36
Cimetidine	-1.52	0.22	8	142	0.6	-1.27	-1.71	-2.44
Ciprofloxacin	-3.57	0.18	20	267	1.58	-3.50	-2.97	-3.34
Cisapride	-6.78	0.17	6	110	3.36	-3.71	-4.30	-4.72
Corticosterone	-3.29	0.17	7	182	2.67	-3.74	-3.80	-3.29
Cortisone Acetate	-4.22	0.13	4	222	2.56	-4.03	-3.89	-4.21
Cyclosporine A	-5.03	0.16	6	151	3.27	-4.03	-7.00	-4.45
Daidzein	-5.23	0.13	5	330	2.87	-5.42	-3.11	-4.47
Desipramine	-3.83	0.18	3	100	3.53	-3.78	-4.14	-4.18
Dexamethasone	-3.56	0.18	16	263	1.9	-3.78	-3.55	-3.80
Diazoxide	-3.43	0.22	4	329	1.87	-4.41	-2.34	-3.16
Diclofenac	-5.34	0.18	34	168	4.36	-5.29	-4.15	-5.35
Diflorasone Diacetate	-4.82	0.16	3	223	2.99	-4.47	-4.20	-4.98
Difloxacin	-3.83	0.21	3	211	2.72	-4.08	-4.05	-4.02
Diltiazem	-3.02	0.13	3	210	3.37	-4.72	-4.24	-4.80
Diphenylamine	-3.53	0.14	3	54	3.43	-3.22	-3.22	-3.68
DOPA,L-	-1.76	0.17	6	270	0.05	-2.00	-1.06	-1.79
Enalapril	-1.36	0.21	3	144	1.6	-2.29	-3.01	-2.90
Estradiol,17α-	-5.00	0.18	5	215	3.61	-5.01	-3.98	-4.78
Estrone	-5.38	0.19	8	255	3.82	-5.62	-4.02	-4.79
Ethoxzolamide	-3.76	0.17	3	189	1.34	-2.48	-2.79	-3.00
Etoposide	-3.60	0.20	4	244	1.34	-3.03	-4.51	-3.52
Eucalyptol	-1.66	0.21	3	37	2.74	-2.36	-2.07	-2.22
Fenbufen	-5.18	0.21	10	186	3.4	-4.51	-3.78	-3.72
Flumequine	-3.90	0.19	3	253	2.35	-4.13	-2.83	-3.76
Flurbiprofen	-4.34	0.20	23	111	3.68	-4.04	-3.64	-4.08
Folic Acid	-5.96	0.16	6	250	-0.04	-1.71	-2.53	-3.58
Ganciclovir	-1.78	0.13	3	250	-1.97	0.22	-0.81	-1.88
Glipizide	-5.61	0.21	9	209	2.08	-3.42	-4.33	-4.68
Griseofulvin	-4.52	0.19	15	220	2.69	-4.14	-3.39	-3.56
Haloperidol	-5.71	0.17	10	151	4.43	-5.19	-4.24	-4.50
Ibrutinib	-4.85	0.19	7	155	4.22	-5.02	-6.43	-5.08
Indinavir	-4.53	0.16	5	168	2.87	-3.80	-5.45	-4.84
Indomethacin	-5.48	0.22	21	159	3.93	-4.77	-4.72	-5.17
Indoprofen	-4.65	0.21	5	214	3.04	-4.43	-3.65	-4.21
Ketoconazole	-5.47	0.14	11	146	4.21	-4.92	-5.95	-5.38
Maprotiline	-4.62	0.22	5	92	4.21	-4.38	-4.53	-4.95
Metolazone	-3.88	0.21	8	256	2.71	-4.52	-4.12	-4.39
Nabumetone	-4.40	0.21	3	80	3.37	-3.42	-3.66	-4.04
Naproxen	-4.23	0.16	17	153	3.04	-3.82	-3.29	-4.08
Nelfinavir	-6.21	0.20	3	350	4.75	-7.50	-5.62	-5.36
Nevirapine	-3.41	0.14	6	248	2.65	-4.38	-3.54	-3.90
Nifedipine	-4.71	0.15	11	173	2.18	-3.16	-3.22	-4.67
Nimesulide	-4.74	0.14	5	144	2.76	-3.45	-3.92	-4.22
Norfloxacin	-2.88	0.16	19	221	1.27	-2.73	-2.67	-3.13
Nortriptyline	-3.93	0.16	5	214	3.83	-5.22	-4.28	-4.51
Noscapine	-4.48	0.14	3	176	2.88	-3.89	-3.95	-3.84
Ofloxacin	-2.03	0.13	14	254	1.54	-3.33	-3.04	-1.37
Oxazepam	-4.03	0.17	5	206	2.45	-3.76	-3.46	-3.65
Oxyphenbutazone	-3.94	0.19	3	96	3.49	-3.70	-3.49	-4.24
Papaverine	-4.33	0.19	12	147	3.86	-4.58	-4.32	-4.42
Perphenazine	-4.48	0.17	6	97	3.94	-4.16	-4.95	-4.74
Phenacetin	-2.30	0.14	10	135	2.04	-2.64	-1.97	-2.14
Phenazopyridine	-4.02	0.16	7	139	2.66	-3.30	-3.10	-3.36
Pindolol	-3.75	0.15	9	170	1.91	-2.86	-2.45	-2.91
Pravastatin	-4.86	0.15	10	326	2.44	-4.95	-3.45	-3.60
Prednisolone, Methyl-	-3.33	0.18	5	233	1.8	-3.38	-3.65	-3.45
Primidone	-2.53	0.14	4	282	0.54	-2.61	-1.97	-2.31
Probenecid	-4.83	0.20	4	197	2.2	-3.42	-2.62	-3.39
Promazine	-4.45	0.13	4	33	4.24	-3.82	-4.33	-4.74
Promethazine	-4.38	0.19	11	60	4.24	-4.09	-4.29	-4.68
Repaglinide	-4.77	0.17	4	131	5.22	-5.78	-5.22	-6.45
Resveratrol, trans-	-3.75	0.18	7	254	2.97	-4.76	-3.04	-3.60
Ritonavir	-5.17	0.16	5	121	5.91	-6.37	-7.47	-5.80
Rofecoxib	-4.61	0.16	5	207	2.56	-3.88	-3.67	-4.11
Spironolactone	-4.21	0.16	6	135	4.85	-5.45	-5.12	-5.25
Strychnine	-3.38	0.19	6	275	2.09	-4.09	-4.06	-3.30
Sulfasalazine	-6.41	0.14	9	220	1.8	-3.25	-3.85	-4.36
Sulfathiazole	-2.62	0.22	9	202	1.53	-2.80	-3.10	-2.57
Sulfisomidine	-2.16	0.14	3	243	1.48	-3.16	-3.21	-2.84
Sulfisoxazole	-3.13	0.14	3	191	1.67	-2.83	-3.09	-2.81
Sulindac	-4.96	0.21	7	184	4.37	-5.46	-4.34	-5.10
Tetracaine	-3.11	0.11	3	149	2.62	-3.36	-2.61	-2.78
Tetracycline	-3.22	0.15	8	165	-0.37	-0.53	-1.68	-2.72
Thiacetazone	-3.50	0.16	10	225	0.81	-2.31	-2.38	-2.80
Triamcinolone	-3.52	0.21	5	270	0.62	-2.57	-3.10	-3.12
Triamterene	-4.11	0.14	9	313	0.83	-3.21	-4.17	-3.42
Warfarin	-4.78	0.20	11	161	3.61	-4.47	-3.84	-4.29
Xanthine	-3.60	0.21	3	300	-1.06	-1.19	-1.24	-2.69
Min.	-6.79	0.11						
Max.	-1.18	0.22						
Mean	-4.03	0.17						

**Table A4.** External Test Set 4 (Avg. Interlab. SD ~0.62)

	log *S*_0_ (avg., 25 °C)			*T*_m_	log *P*	Calculated log *S*_0_		
NAME	(*Wiki-pS_0_*)	SD	n	(°C)	(RDKit)	GSE	ABSOLV	RFR
Amantadine	-2.19	0.50	3	180	1.91	-2.96	-1.95	-1.96
Amiodarone	-10.40	0.50	5	156	6.94	-7.75	-7.68	-6.77
Amodiaquine	-5.49	0.65	3	208	5.18	-6.51	-4.86	-5.57
Bisoprolol	-2.09	0.59	3	100	2.37	-2.62	-2.51	-2.50
Bromocriptine	-5.50	0.51	5	217	3.19	-4.61	-5.65	-5.12
Buprenorphine	-6.07	0.83	3	210	4.41	-5.76	-5.54	-5.20
Chlorprothixene	-5.99	0.51	6	98	5.19	-5.42	-4.96	-5.23
Clofazimine	-9.05	0.93	5	211	7.49	-8.85	-7.42	-6.88
Curcumin	-5.36	0.68	3	177	3.37	-4.39	-4.22	-4.67
Danazol	-6.10	0.52	10	229	4.22	-5.76	-5.01	-4.69
Didanosine	-1.24	0.54	3	162	-0.21	-0.66	-1.75	-1.34
Diflunisal	-4.99	0.56	11	214	3.04	-4.43	-3.70	-4.02
Diphenhydramine	-3.21	0.55	4	169	3.35	-4.29	-3.47	-3.41
Etoxadrol	-1.96	0.55	3	*124*	2.81	-3.30	-2.96	-3.14
Ezetimibe	-4.94	0.51	4	165	4.89	-5.79	-4.62	-5.55
Fentiazac	-5.84	0.65	4	161	4.76	-5.62	-5.40	-4.90
Iopanoic Acid	-5.49	0.66	3	155	3.74	-4.54	-5.94	-4.85
Itraconazole	-8.98	0.61	3	165	5.58	-6.48	-8.45	-6.50
Miconazole	-5.82	0.50	6	161	6.45	-7.31	-5.86	-5.71
Mifepristone	-5.22	0.75	4	194	5.41	-6.60	-5.96	-5.82
Omeprazole	-3.70	0.50	3	156	2.9	-3.71	-3.70	-3.88
Pioglitazone	-6.20	0.66	4	184	3.16	-4.25	-4.15	-4.44
Procaine	-2.30	0.60	3	61	1.77	-1.63	-2.27	-2.55
Quinine	-3.06	0.57	7	177	3.17	-4.19	-3.74	-2.85
Raloxifene	-6.82	0.56	6	145	6.08	-6.78	-6.70	-6.18
Rifabutin	-4.09	0.66	3	*176*	4.62	-5.63	-6.88	-5.22
Saquinavir	-5.92	0.58	3	350	3.09	-5.84	-5.95	-4.92
Sulfadimethoxine	-3.74	0.70	3	204	0.88	-2.17	-2.89	-3.17
Tamoxifen	-7.52	0.72	7	98	6.00	-6.23	-5.55	-6.09
Telmisartan	-6.73	0.84	5	262	7.26	-9.13	-9.03	-7.15
Terfenadine	-7.74	0.71	11	150	6.45	-7.20	-6.03	-6.61
Thiabendazole	-3.97	0.50	4	305	2.69	-4.99	-3.75	-3.62
Min.	-10.40	0.50						
Max.	-1.24	0.93						
Mean	-5.24	0.62						
